# The clinical relevance of formal thought disorder in the early stages of psychosis: results from the PRONIA study

**DOI:** 10.1007/s00406-021-01327-y

**Published:** 2021-09-17

**Authors:** Oemer Faruk Oeztuerk, Alessandro Pigoni, Julian Wenzel, Shalaila S. Haas, David Popovic, Anne Ruef, Dominic B. Dwyer, Lana Kambeitz-Ilankovic, Stephan Ruhrmann, Katharine Chisholm, Paris Lalousis, Sian Lowri Griffiths, Theresa Lichtenstein, Marlene Rosen, Joseph Kambeitz, Frauke Schultze-Lutter, Peter Liddle, Rachel Upthegrove, Raimo K. R. Salokangas, Christos Pantelis, Eva Meisenzahl, Stephen J. Wood, Paolo Brambilla, Stefan Borgwardt, Peter Falkai, Linda A. Antonucci, Nikolaos Koutsouleris, the PRONIA Consortium

**Affiliations:** 1 Department of Psychiatry and Psychotherapy, Ludwig-Maximilian-University Munich, Nussbaumstr. 7, 80336 Munich, Germany; 2 International Max Planck Research School for Translational Psychiatry, Munich, Germany; 3 Max Planck Institute for Psychiatry, Munich, Germany; 4 MoMiLab Research Unit, IMT School for Advanced Studies Lucca, Lucca, Italy; 5 Department of Psychiatry and Psychotherapy, Faculty of Medicine and University Hospital of Cologne, University of Cologne, Cologne, Germany; 6 Department of Psychiatry, Icahn School of Medicine at Mount Sinai, New York, USA; 7 School of Psychology, Aston University, Birmingham, UK; 8 Institute for Mental Health, University of Birmingham, Birmingham, UK; 9 Department of Psychiatry and Psychotherapy, Medical Faculty, Heinrich-Heine University, Düsseldorf, Germany; 10 Division of Psychiatry and Applied Psychology, Institute of Mental Health, University of Nottingham, Nottingham, UK; 11 Department of Psychiatry, University of Turku, Turku, Finland; 12 Melbourne Neuropsychiatry Centre, University of Melbourne, Melbourne, Australia; 13 Melbourne Health, Melbourne, Australia; 14 School of Psychology, University of Birmingham, Birmingham, UK; 15 Orygen, The National Centre of Excellence for Youth Mental Health, Melbourne, Australia; 16 Centre for Youth Mental Health, University of Melbourne, Melbourne, Australia; 17 Department of Neurosciences and Mental Health, Fondazione IRCCS Ca’ Granda Ospedale Maggiore Policlinico, University of Milan, Milan, Italy; 18 Department of Psychiatry, University Psychiatric Clinic, Psychiatric University Hospital, University of Basel, Basel, Switzerland; 19 Department of Basic Medical Sciences, Neuroscience and Sense Organs, University of Bari “Aldo Moro”, Bari, Italy; 20 Department of Education, Psychology and Communication Science, University of Bari “Aldo Moro”, Bari, Italy; 21 Department of Psychosis Studies, Institute of Psychiatry, Psychology and Neuroscience, King’s College London, London, UK

**Keywords:** Formal thought disorder, Early psychosis, Clustering, Functioning, Neurocognition

## Abstract

**Background:**

Formal thought disorder (FTD) has been associated with more severe illness courses and functional deficits in patients with psychotic disorders. However, it remains unclear whether the presence of FTD characterises a specific subgroup of patients showing more prominent illness severity, neurocognitive and functional impairments. This study aimed to identify stable and generalizable FTD-subgroups of patients with recent-onset psychosis (ROP) by applying a comprehensive data-driven clustering approach and to test the validity of these subgroups by assessing associations between this FTD-related stratification, social and occupational functioning, and neurocognition.

**Methods:**

279 patients with ROP were recruited as part of the multi-site European PRONIA study (Personalised Prognostic Tools for Early Psychosis Management; www.pronia.eu). Five FTD-related symptoms (conceptual disorganization, poverty of content of speech, difficulty in abstract thinking, increased latency of response and poverty of speech) were assessed with Positive and Negative Symptom Scale (PANSS) and the Scale for the Assessment of Negative Symptoms (SANS).

**Results:**

The results with two patient subgroups showing different levels of FTD were the most stable and generalizable clustering solution (predicted clustering strength value = 0.86). FTD-High subgroup had lower scores in social (*p*_fdr_ < 0.001) and role (*p*_fdr_ < 0.001) functioning, as well as worse neurocognitive performance in semantic (*p*_fdr_ < 0.001) and phonological verbal fluency (*p*_fdr_ < 0.001), short-term verbal memory (*p*_fdr_ = 0.002) and abstract thinking (*p*_fdr_ = 0.010), in comparison to FTD-Low group.

**Conclusions:**

Clustering techniques allowed us to identify patients with more pronounced FTD showing more severe deficits in functioning and neurocognition, thus suggesting that FTD may be a relevant marker of illness severity in the early psychosis pathway.

**Supplementary Information:**

The online version contains supplementary material available at 10.1007/s00406-021-01327-y.

## Introduction

Psychotic disorders are closely linked with neurocognitive and functional impairments [17, 49] that frequently precede disease onset and persist after remission of the acute illness [22, 37]. Formal thought disorder (FTD) is a multifaceted construct of disturbances in thought, communication and language, such as loosening of associations, blocking, semantic and phonemic paraphasia [28, 42]. Previous literature revealed that FTD is not only a core feature of psychosis but that it is also associated with adverse social and functional outcomes in psychotic patients [3, 21, 25, 27, 28]. More specifically, FTD is associated with an increased (re-)hospitalization rate [44], unemployment risk [34], reduced quality of life [48] and adjustment abilities indexed by occupational functioning and self-support [34]. Harrow et al. [20] highlighted that patients with schizophrenia experiencing enduring FTD after the acute phase of psychosis showed lower occupational functioning levels and higher relapse/re-hospitalisation rates. Moreover, thought and communication disturbances in youth at clinical high risk for psychosis were associated with reduced social and occupational functioning outcomes and a higher risk for transition to the established disease [46]. Furthermore, cognitive basic symptoms including subjective thought blockage, interference and pressure as well as disturbances of abstract thinking and expressive and receptive speech that might be regarded as subclinical presentations of observable FTD have been demonstrated to predict subsequent psychosis [45]. In summary, these findings may point towards FTD playing an important role in explaining the behavioural, psychopathological and functional heterogeneity of disease manifestations in both early and prodromal phases of psychosis. So far, this aspect has remained under-investigated, [42, 43] as research on clinical markers of psychosis has focused rather on positive and negative symptoms as well as on cognitive phenotypes of the disorders [44]. However, this traditional approach may not fully capture the clinical heterogeneity of psychosis in terms of both disease course and severity [21, 24, 27, 42]. FTD may represent a clinical fingerprint of disease severity [42] as it encompasses observable speech-related, and cognitive impairments of psychosis. As findings from a recent systematic review indicated, FTD—especially disorganization—might have early diagnostic and prognostic relevance in the early stages of psychosis [39]. The main findings of this systematic review showed that FTD severity predicted poor social functioning, unemployment, relapses, rehospitalisations, as well as correlations between attentional deficits, executive functions and FTD severity, and highlighted the predictive potential of executive dysfunctions for sustained FTD. Therefore, FTD stratification could help clinician to detect a subgroup of patients at risk of developing poor disease outcomes, who may need early preventive interventions targeting specifically FTD-related deficits.

Recently, there has been great interest in machine learning and pattern recognition techniques, which have shown to be promising tools for addressing clinical heterogeneity in psychiatric disorders [7, 15, 35]. Among these, unsupervised clustering algorithms allow us to explore the subgroup structure of psychopathological phenomena in a quantitative and potentially unbiased way [23]. Using unsupervised machine learning techniques to investigate the role of FTD role in the heterogeneity of psychosis phenotypes would allow (i) identifying more homogeneous clinical subgroups experiencing differential illness manifestations, and (ii) exploring the interdependence of possible FTD clusters with other phenotypic expressions of psychosis.

Thus, this study aims for the first time (1) to evaluate whether it is possible to identify robust subtypes of patients with recent-onset psychosis (ROP) that are characterized by distinct FTD patterns, (2) to investigate whether this FTD-related stratification is associated with clinical (i.e., the Global Social (GF-Social) and Role (GF-Role) Functioning), and neurocognitive (i.e., Wechsler Adult Intelligence Scale (WAIS-III; premorbid verbal intelligence), Phonological and Semantic Verbal Fluency (VF—P & S), and Auditory Forward and Backward Digit Span (ADS—Forward and Backward) phenotypes at an early stage of the disease, and (3) to explore the potential associations between FTD-related symptoms, functioning and neurocognition.

## Methods

The PRONIA (Personalised Prognostic Tool for Early Psychosis Management) study recruited patients into the recent-onset psychosis (ROP) study group if, i) they fulfilled the DSM-IV-TR criteria for affective and non-affective psychotic episode lifetime, ii) the psychotic episode was present within the past 3 months, and iii) the onset of psychosis occurred within the past 24 months. Exclusion criteria were treatments with antipsychotic medication for longer than 90 days (cumulative number of days) at or above minimum dosage of the 1st episode psychosis range of (DGPPN) S3 guideline. [16] General inclusion and exclusion criteria are detailed in the Supplementary Sect. 1. Based on these criteria, we were able to analyse clinical and neurocognitive data from 279 individuals experiencing a ROP between 15 and 40 years of age (Table 1). Patients were recruited by the PRONIA Consortium between January 2014 and December 2017 at ten sites across five countries (Table 1). All adult participants gave their written informed consent prior to study inclusion. Participants younger than 18 years provided written, informed assent, and their caregivers written, informed consent before being enrolled in the study.

1. Psychopathological assessment of the severity of formal thought disorder

**Table 1:** Study-associated sociodemographic

	Formal thought disorder related symptom severity			
	Low	High	$\chi^{2}$	*p* value
Age, median	24	23		0.011
Female, No. (%)	91 (44)	28 (37)	0.7053	0.401
Education year, median	14	12		< 0.001
Participants per site, No				
The Ludwig-Maximilian-University Munich	76	20	16.452	0.058
The University of Cologne	30	15		
The University of Münster	6	4		
The University of Düsseldorf	2	3		
The University of Basel	18	6		
The University of Turku	33	7		
The University of Milan	15	10		
The University of Udine	8	3		
The University of Bari	1	3		
The University of Birmingham	16	3		

The FTD severity has been assessed with different scales [1, 8, 14] since Kraepelin and Bleuer postulated the importance of earlier manifestation of this clinical phenomenon in an evolving psychosis [21]. The Thought and Language disorder (TALD) scale from Kircher et al. [28] is a validated instrument for assessing FTD. Indeed, it allows clinicians to examine the multifaceted nature of FTD and distinguish positive and negative thought disorder with subjective and objective components. We operationalized only observed positive and negative FTD with items from the Positive and Negative Symptom Scale (PANSS) [26] and the Scale for the Assessment of Negative Symptoms (SANS) [2] with a psychopathological orientation on the Thought and Language disorder (TALD) scale from Kircher et al. [28]

More in detail, observed positive FTD was assessed through:

- conceptual disorganization (PANSS P2) item, reflecting the following psychopathological alterations listed in the TALD scale; tangentiality, circumstantiality, derailment, dissociation of thinking, cross talk, and logorrhoea [26, 28].
- poverty of content speech (SANS 10) item is included in the TALD scale [2, 28]
  On the other hands, observed negative FTD was assessed through:
- the difficulty in abstract thinking (PANSS N5) item is called as concretism in the TALD scale, that reflects the same psychopathological alteration [26, 28].
- increased latency of response (SANS 12) item is called as slowed thinking in the TALD scale that also reflects the same psychopathological alteration [2, 28].
- poverty of speech (SANS 9) item is included in the TALD scale [2, 28].

These five FTD-related symptoms were used as features to cluster patients with ROP into FTD subgroups. Notably, PANSS N6 item (i.e., “Lack of spontaneity and flow of conversation”) and SANS 11 item (i.e., “Blocking”) individual scores reflecting following TALD scale subjective negative items: “Poverty of thought”, “Dysfunction of thought initiative”, “Intentionality and expressive speech dysfunction” and “Inhibited thinking”, were not included as a feature on purpose in the present study to avoid redundancy between objective and subjective FTD assessments [28, 33].

Trained clinicians assessed psychopathology of each participant and interrater reliability tests were performed regularly to calibrate PANSS [Intraclass Correlation (ICC) = 0.79] across study sites. Interrater reliability test for SANS is not available.

2) Clustering FTD subgroups based on psychopathological patterns

Our first aim was to identify a stable and generalizable ROP patient stratification based on distinct patterns of FTD identified by means of data-driven unsupervised machine learning techniques. After scaling the data (Supplementary Sect. 2), we applied the following steps to investigate and compare alternative FTD subgroup solutions.

- 2a) Validity and stability of clustering methods

First, we used the *ClValid* [6] package to assess three clustering algorithms: (i) k-means, (ii) hierarchical clustering, and (iii) partitioning around medoids (PAM) with the number of clusters ranging from 2 to 10. *ClValid* [6] compares solutions from several clustering algorithms while the numbers of clusters vary. This allows researchers to choose the optimal algorithm and number of clusters with a majority rule based on a battery of internal validity and stability measures (for a full description, see [6]). We tested average and ward linkage for the hierarchical clustering due to previous simulation study from Walesiak and Dudek [54] reporting them as the best linkages for ordinal data. The average linkage considers the distance between two clusters as the average distance between each point in one cluster to every point in the other cluster, whereas ward linkage is a method that minimizes the error sum of squares between the clusters over all the variables. We selected the optimal algorithms and numbers of clusters by applying a majority rule among computed internal validity and stability measures (Supplementary Sect. 3).

Second, we retested the winner algorithms determined with majority rule in the first step with the *NbClust* [11] package for the optimal number of clusters (Supplementary Sect. 3). Many indices have been reported previously to decide for a valid clustering solution in a given dataset. *NbClust* provides an automatized scan through 26 validity indices such Calinski–Harabasz (CH) Index [9], Davies–Bouldin (DB) Index [13], Silhouette Index [46] and reports the optimal number of clusters for a given clustering algorithm with majority rule. Following the first step, we tested the average and ward linkage for the hierarchical clustering algorithm, which was one of the winner algorithms (Supplementary Sect. 3).

- 2b) Stability and generalizability of the optimal cluster solutions

Given that the first and second steps resulted in more than one optimal solution, namely k-means and hierarchical algorithms (Supplementary Sect. 3), we inspected the solutions from the hierarchical clustering for their clinical validity and robustness with the figure for silhouette width as well as the cophenetic correlation in a third step (Supplementary Sect. 4).

Thereafter, we examined the stability and generalizability of k-means algorithm solution selected based on the previous steps (Supplementary Sect. 5 and 6). To exclude possible package or function-specific effects, we retested the robustness of the k-means-based clustering solution with the R package *ClusterStability* [31]. This package allows screening popular validity measures and provides researchers with a global stability (ST) index and an individual ST-index ranging from 0 to 1, where 1 indicates very strong stability (Supplementary Sect. 5).

Lastly, we investigated the predicted clustering strength of the partitioning algorithms using the *predict.strength* package of Tibshirani et al. [52], which applies the principles of cross-validation, well established in supervised machine learning, to the unsupervised case. *Predict.strength* performs n-fold random resampling, partitions the resampled population into training and test data folds, and clusters these with varying numbers of clusters through m iterations. For each iteration, the centroids of training data are applied to test data to compute the proportion of pair–observations falling into the same cluster with the centroids of test data. In the present study, subjects were randomly resampled over 500 iterations and partitioned each time into test and training datasets using two-fold cross-validation. The highest prediction strength over cut off value 0.80 was chosen as the optimal *predict.strength* value, following published procedures [52] (Supplementary Sect. 6).

The k-means clustering solution survived these validity, stability and generalizability tests and was chosen for further association tests with global and syndromal measures of disease severity, such as the PANSS and SANS total scores, as well as each PANSS and SANS subscales between the identified FTD subgroups. Respective results were reported in Supplementary Sect. 7. To test the specificity of the k-means clustering solution, we run several sanity analyses using items from PANSS and SANS subscales that are not related to FTD and reported the results in Supplementary Sect. 8.

**Fig. 1:**
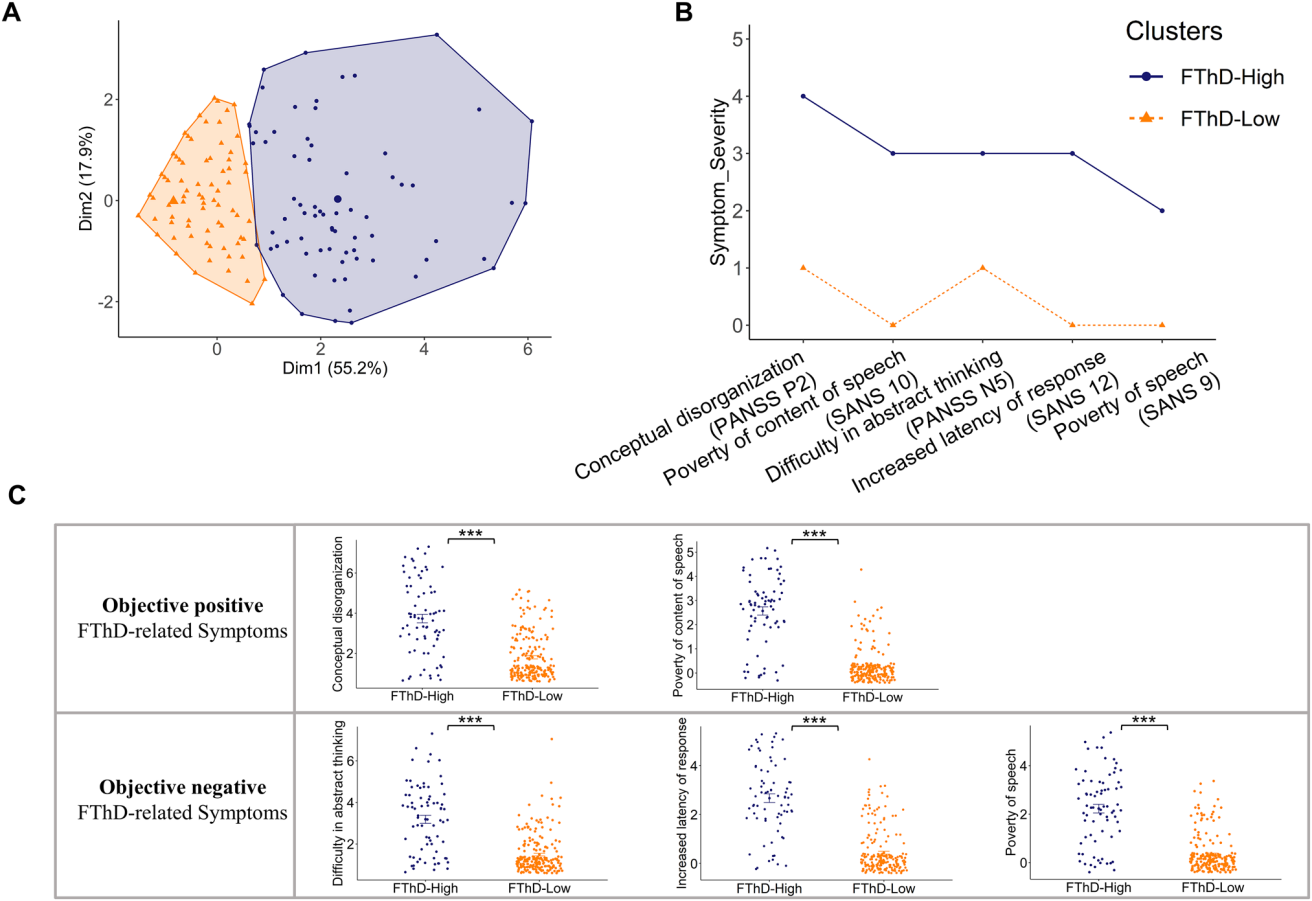
The Psychopathological Comparison of FTD subgroups. **A** Represents the results of the principal component analysis in two-dimensional space, **B** the difference between medians of FTD-related symptom severity, **C** the distributions of each FTD-related symptom and their statistical comparisons with Wilcoxon rank-sum test. Statistical significances are shown ***p_fdr_ < 0.001

**Fig. 2:**
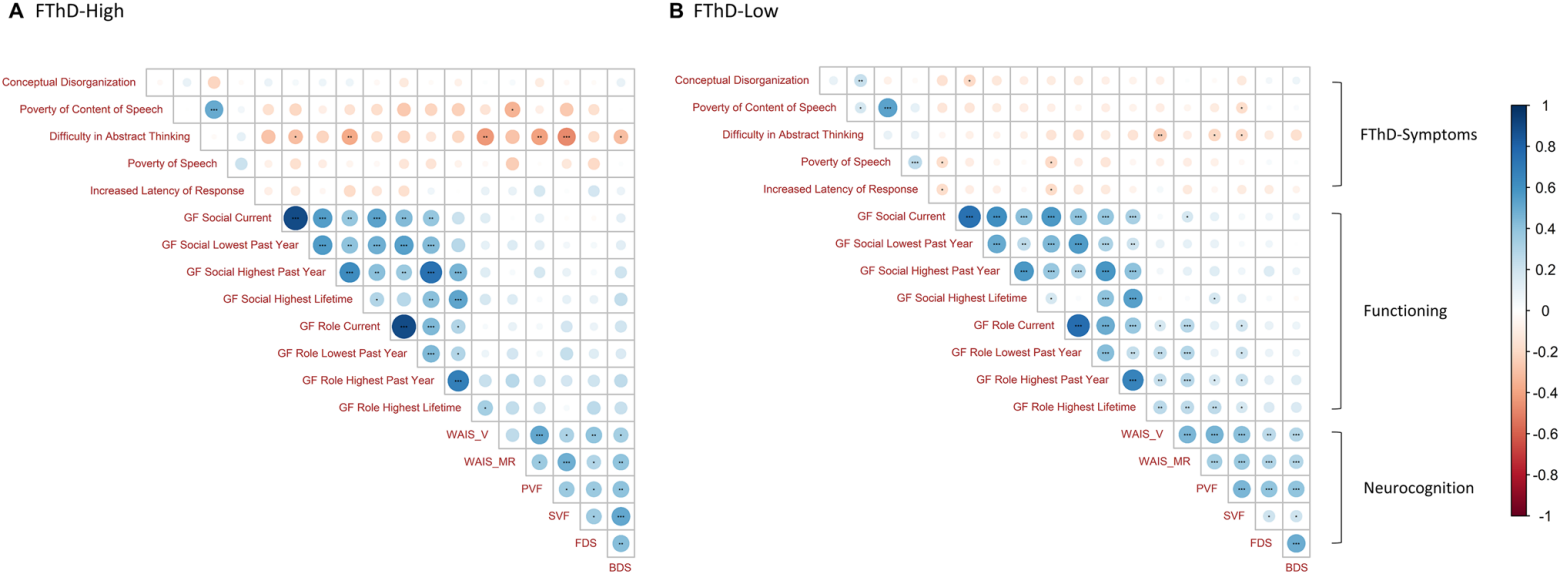
Correlogram among FTD-related symptoms, functioning and neurocognition; **A** FTD-High **B** FTD-Low. Different colours: red = negative or blue = positive represent the direction of correlations, different size of the circles represents the strength of the correlations. Statistical Significances are shown; *p_fdr_ < 0.05, **p_fdr_ < 0.01, ***p_fdr_ < 0.001

We compared age, educational years, clinical outcomes; Global Social (GF-Social) and Role (GF-Role) Functioning scores, and neuropsychological performances; abstract reasoning, verbal fluency, processing speed, verbal short-term and working memory between FTD subgroups using the Mann–Whitney-*U* test [32] after checking for normal distribution with the Shapiro–Wilk test [48]. Distributions of sex, site and FTD subgroups, respectively, were compared using the $\chi^{2}$ tests [36] (Tables 1 and 2). All analyses and univariate statistical comparisons were conducted with R version 3.5.2. We used the False Discovery Rate (FDR) [5] to correct all *P* values for the multiple comparisons. *P* values of the Sociodemographic: Age, sex, education, and the number of participants per site, *P* values of the clinical outcomes and *P* values of the neurocognition were considered dependent and corrected using FDR. We provided effect sizes calculated with the *wilcoxonR* function in the R package *rcompanion* for each nonparametric statistical comparison (Fig. 1). We tested the FDR-corrected significance of correlations and provided the correlogram that is a graphical representation of the correlation matrix of all included variables. (Fig. 2).

- 3a) Association of the clustering solution with clinical outcomes; social and role functioning

**Table 2:** Clinical, functioning and neurocognition differences in individuals with recent-onset psychosis

Characteristics	Formal thought disorder related symptom severity			
	Low	High	*p* value	*p*_fdr_ value
Global functioning Social scale rated at baseline				
Highest lifetime score, median	8	8	< 0.001	< 0.001
Highest score in past year, median	7	6	< 0.001	< 0.001
Lowest score in past year, median	5	5	0.005	0.005
Current score, median	6	5	< 0.001	< 0.001
Global functioning Role scale rated at baseline				
Highest lifetime score, median	8	8	< 0.001	< 0.001
Highest score in past year, median	7	6	< 0.001	< 0.001
Lowest score in past year, median	5	4	0.001	0.002
Current score, median	6	5	< 0.001	< 0.001
Neurocognition at baseline				
WAIS—premorbid verbal intelligence, median	10	9	0.001	0.002
WAIS—Matrices, median	10	9	0.008	0.010
Phonological Verbal Fluency, median	13	11	< 0.001	< 0.001
Semantic Verbal Fluency, median	21	16	< 0.001	< 0.001
Forward Digit Span, median	9	8	< 0.001	0.002
Backward Digit Span, median	6	6	0.015	0.015

We investigated the association of the selected clustering solution with clinical outcomes by performing between-groups statistical comparisons of baseline the GF-Social and GF-Role functioning scores including the retrospective assessments of highest functioning levels lifetime as well as past year. [10, 12] Trained clinicians assessed the functioning level of each participant and interrater reliability tests were performed regularly to calibrate the Global Functioning scales; GF-Social [Intraclass Correlation (ICC) = 0.945 and GF-Role (ICC = 0.924)] across study sites [29]. We compared the differences in social and role functioning scores between two clusters with the Mann–Whitney-*U* test [32] or with the Welch’s two-sample *t*-test [54] after checking for normal distribution with the Shapiro–Wilk test [48].

- 3b) Association of FTD-defined subgroups and neurocognitive performance

We analysed the following neurocognitive domains; the WAIS-III; premorbid verbal intelligence assessing visual processing and abstract reasoning, the VF—P & S assessing verbal fluency and processing speed and the ADS—F & B assessing verbal short-term memory and verbal working memory from the neurocognitive battery of the PRONIA study (Supplementary Table 1), because previous literature has shown a significant association between these neurocognitive domains and FTD as measured by Thought, Language, and Communication (TLC) [1] and TALD [28] scale [38, 41, 42, 51, 53]. Welch’s two-sample *t*-test [55] or Mann–Whitney-*U* test [32] (based on the results of the Shapiro–Wilk test [48]) were used to assess differences between FTD subgroups in their neurocognitive performances.

We ran several sanity analyses with the following aims: (1) to test whether our clustering solution was associated with global and syndromal disease severity, (2) to investigate the interaction between FTD-related symptoms, (3) to corroborate the specificity of our clustering solution with FTD-related symptoms, (4) to exclude that the clustering algorithms provide us with a solution more prone to detect negative symptom pattern and (5) to test whether our clustering protocol recognizes a positive symptom pattern, and reported results in the Supplementary Sects. 7 and 8 in details. These sanity analyses showed us that our FTD-driven clustering solution was clinically valid and specific to FTD-related symptom severity. Analyses with non-FTD items from PANSS and SANS as well as their subscale showed less stable and less generalizable clustering solutions than the FTD-driven clustering solution.

## Results

Our multi-step clustering analyses identified a k-means algorithm-based two-cluster solution as the most stable and generalizable stratification approach. This approach delineated two FTD subgroups, FTD-High and FTD-Low (*n* = 75 vs. 204) in our ROP patient cohort (Fig. 1). The clustering solution was significantly informed by (1) observed positive FTD as measured by conceptual disorganization (PANSS P2) (median = 4 vs. 1, *p*_fdr_ < 0.001, *r* = 0.488) and poverty of content of speech (SANS 10) (median = 3 vs. 0, *p*_fdr_ < 0.001, r = 0.712), and (2) observed negative FTD as measured by difficulty in abstract thinking (PANSS N5) (median = 3 vs. 1, *p*_fdr_ < 0.001, *r* = 0.503), increased latency of response (SANS 12) (median = 3 vs. 0, *p*_fdr_ < 0.001, r = 0.653) and poverty of speech (SANS 9) (median = 2 vs. 0, *p*_fdr_ < 0.001, *r* = 0.611) (Fig. 1).

As reported in Table 1, we observed no significant interaction of FTD-informed subgroups with sex ($\chi^{2}$ = 0.7053, *p*_fdr_ = 0.401, phi = 0.050) and site ($\chi^{2}$ = 16.452, *p*_fdr_ = 0.073, phi = 0.243). Furthermore, the distribution of missing data did not show significant differences between FTD subgroups (Supplementary Table 5). At baseline, the FTD-High group was younger than the FTD-Low group (median = 23 vs. 24, *p*_fdr_ = 0.022, *r* = 0.153) in group level comparison (Supplementary Fig. 5). The two groups also differed in their education level: with fewer years in education in FTD-High group than in FTD-Low group (median = 12 vs. 14, *p*_fdr_ = 0.002, *r* = 0.209). Furthermore, education years and age were positively correlated in each subgroup: FTD-High (*p* < 0.001, *r* = 0.51) and FTD-Low (*p* < 0.001, *r* = 0.36).

Cluster assignment was not influenced by global disease severity as measured by PANSS total score at baseline (*p*_fdr_ = 0.779, *r* = 0.017) but was associated with more pronounced negative symptoms as measured by SANS total score at baseline (*p*_fdr_ < 0.001, *r* = − 0.551) in the high FTD subgroup (Supplementary Table 6).

### Comparison of functioning between FTD subgroups

**Fig. 3:**
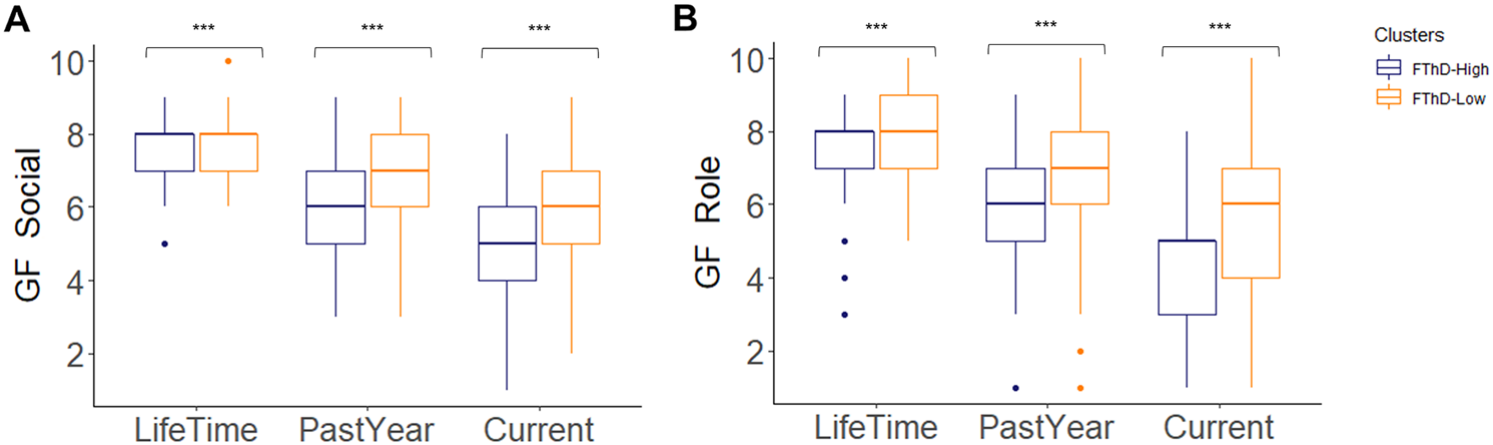
The Comparison of functioning levels in social and role functioning domains. Statistical comparisons are conducted with the Welch two-sample or the Mann–Whitney-*U* tests based on the distribution of the data. Statistical Significances are shown; ***p_fdr_ < 0.001

FTD subgroups differed significantly in the GF-Social and GF-Role instruments (Table 2 and Fig. 3). The GF-Social scores were lower in the FTD-High compared to the FTD-Low group in the highest lifetime (*p*_fdr_ < 0.001, *r* = − 0.216), past year (*p*_fdr_ < 0.001, *r* = − 0.219) and baseline variables (*p*_fdr_ < 0.001, *r* = − 0.269). Similarly, GF-Role scores were lower in the FTD-High vs. FTD-Low group in the highest lifetime (*p*_fdr_ < 0.001, *r* = − 0.229), past year (*p*_fdr_ = 0.001, *r* = − 0.242) and baseline variables (*p*_fdr_ < 0.001, *r* = − 0.259).

### Comparison of neurocognitive performance between FTD subgroups

Comparisons of neurocognitive measures between FTD subgroups showed significant differences in verbal and semantic fluency, verbal short-term memory and abstract reasoning (Table 2). The WAIS-Vocabulary (*p*_fdr_ = 0.002, *r* = − 0.200) and WAIS-Matrices (*p*_fdr_ = 0.010, *r* = − 0.166) scores were lower in the FTD-High group than in the FTD-Low group. We found a similar pattern of results in the phonological verbal fluency (*p*_fdr_ < 0.001, *r* = − 0.235) and semantic fluency (*p*_fdr_ < 0.001, *r* = − 0.326) scores, as well as in the forward (*p*_fdr_ = 0.002, *r* = − 0.204) and backward (*p*_fdr_ = 0.015, *r* = − 0.151) digit span scores, i.e., FTD-High individuals always performed worse than FTD-Low group in these neurocognitive domains.

### Correlation analyses of FTD-related symptoms, functioning and neurocognition

The correlation analyses in the FTD-High subgroup revealed that “difficulty in abstract thinking” correlated negatively with functioning GF-Social lowest in past year: (*p*_fdr_ = 0.038, *r* = − 0.30); GF-Social highest in lifetime: (*p*_fdr_ = 0.006, *r* = − 0.37)), and neurocognitive domains (WAIS-Vocabulary: (*p*_fdr_ = 0.002, *r* = − 0.43); backward digit span: (*p*_fdr_ = 0.042, *r* = − 0.30); phonological verbal fluency: (*p*_fdr_ = 0.003, *r* = − 0.40); and semantic fluency: (*p*_fdr_ < 0.001, *r* = − 0.48)). Poverty of content of speech item was significantly associated with WAIS-Matrices (*p*_fdr_ = 0.016, *r* = − 0.35) in the FTD-High subgroup. The correlations between other FTD-related symptoms and other variables in functioning or neurocognition were either non-significant or significant with a small effect size (*r* < 0.30) in both subgroups (Fig. 2).

## Discussion

In this multisite naturalistic study, we addressed for the first time the psychopathological heterogeneity of patients with recent-onset psychosis from the perspective of the core syndrome of FTD with unsupervised machine learning algorithms. Furthermore, we investigated the broader quantitative associations between FTD, neurocognitive performance and level of functioning in the early stage of psychosis with group-level statistical comparisons. Our multi-step clustering analysis yielded a solution with two FTD subgroups as the most optimal stratification scheme after running the following four checkpoints; (i) validity, (ii) re-evaluation of validity results and unbiased determination of the winning algorithm, (iii) stability test and (iv) generalizability test for the best clustering solution. In summary, the winning clustering solution revealed two stable subgroups of patients with high and low severity of FTD, which were independent of global disease severity in this early stage of the disease. The FTD-High subgroup showed significant impairment of all functional domains and significantly lower neurocognitive performance in verbal and semantic fluency, short-term verbal memory and abstract thinking. Moreover, the median age at baseline was one year less in both sexes in the FTD-High subgroup and an earlier differentiating peak of male distribution was observed in the FTD-High subgroup in the late adolescence and early adulthood. (Supplementary Fig. 5) This is in keeping with previous clinical observations of worse prognostic long-term outcomes in males. [19, 30]

Our results are also in line with the previous literature showing that thought disorders are negatively associated with role functioning [20], and thought and communication disturbances are related to poorer social and role functioning levels [4]. Retrospective clinical assessments at study inclusion indicated that FTD subgroups started to deviate in symptom and disability measures as well as in social and role functioning already in the year prior to the study. The more specific assessment of social and role functioning differences between FTD subgroups extended this observation to the lifetime scale that may point to FTD subtypes being as a sensitive prognostic marker for a later manifestation of psychosis. [4, 18, 56] These findings may represent a starting point for further investigation of these alterations that may help in identify an early diagnostic and interventional window in which interventions focusing on FTD-related impairments may be beneficial in improving functioning during the early stages of psychosis.

Furthermore, we found that FTD stratification was associated with reduced verbal and semantic fluency, and impairments in short-term verbal memory and abstract thinking. This association between FTD and neurocognitive performance in semantic processing, executive functioning, abstract thinking adds to the previous literature. [38, 41, 42, 50, 52] We may speculate that the lower neurocognitive performance of FTD-High group could be seen from a causal consequential perspective and may drive the impaired social and occupational functioning in this subgroup requiring regular and frequent follow-up examinations. [40, 57] However, further studies addressing this interrelation between FTD and neurocognitive performance are warranted to validate this speculation. Moreover, future studies should be conducted to understand whether the relationship between cluster assignment and neurocognition is moderated or mediated by the severity of negative symptoms.

### Limitations

A validated specific scale to assess FTD and SAPS (Scale for the Assessment of Positive Symptoms) are missing in the presented study. We assessed a restricted part of FTD spectrum with the overlapping symptoms from PANSS and SANS that mapped on to the TALD. This reduces the complexity of the possible interpretations of our results. Therefore, our findings should be considered as preliminary, and further studies employing a more thorough investigation of FTD manifestations through much more assessments (i.e., TALD, SAPS) are warranted to understand the degree of replicability of our findings. Moreover, the presented study did not differ affective and non-affective psychosis. Another limitation is the cross-sectional nature of the analysed data from the baseline examinations, which significantly limits any causal speculation. Lastly, a missing external validation sample restricts the generalizability of these clustering solutions. Further studies applying presented stratification scheme at the single patient level in an external sample are neededQuery.

### Conclusions

The presented multi-step clustering study demonstrated subgroups of patients with distinct clinical presentations of FTD who may have divergent preventive and therapeutic needs related to differential FTD severity. Our findings elucidate how unsupervised machine learning techniques may provide novel insight about the associations between psychopathology, neurocognition and functioning.

In summary, our findings suggest that FTD may be a relevant marker of illness severity in the early psychosis pathway. The reciprocal associations between FTD, functional, and neuropsychological phenotypes of psychosis emphasize the importance of specific treatment pathways for people with more severe FTD. Furthermore, they highlight how FTD may potentially represent a target variable for individualized psycho-, socio-, logotherapeutic interventions aimed at improving neurocognition abilities and personal functioning. Prospective studies should further test this promising perspective.

## Supplementary Information

Below is the link to the electronic supplementary material.

Supplementary file1 (DOCX 2726 KB)
