# Supplementary material for: The clinical relevance of formal thought disorder in the early stages of psychosis: results from the PRONIA study
Source: Eur Arch Psychiatry Clin Neurosci. 2021 Sep 17;272(3):403–13. doi: 10.1007/s00406-021-01327-y (PMC8938366; doi:10.1007/s00406-021-01327-y)
Supplement: Supplementary file 1 — Supplementary file1 (DOCX 2726 KB) [file 406_2021_1327_MOESM1_ESM.docx]

**The clinical relevance of formal thought disorder in the early stage of psychosis:**

**results from PRONIA - Cohort**

Supplementary Material

List of Contents:

1. Inclusion and Exclusion Criteria and general methodological information for the PRONIA study
2. Scaling Data
3. The decision for k-means clustering and re-evaluation of the clustering stability
4. Exclusion the hierarchical clustering solutions
5. The re-evaluation of the cluster stability
6. The generalizability of the cluster solution
7. Comparison of psychopathological subdomains of clusters
8. The cluster solution specificity and sanity analyses
9. Comparison of neurocognitive performances corrected for years of education
10. Supplementary Tables 1 - 7
11. Supplementary Figures 1 - 10

**1. Inclusion and Exclusion Criteria and general methodological information for the PRONIA study**

The Personalized Prognostic Tool for Early Psychosis Management (PRONIA) study was registered at the German Clinical Trials Register (DRKS00005042) and the local research ethics committees at each collaboration site approved the study. (Koutsouleris et al., 2018) The PRONIA study included participants with age between 15 and 40 years, with sufficient language skills for participation and providing informed consent/assent. MRI compatibility is another general inclusion criterion due to the main neuroimaging aspect of the study. Individuals are excluded if they have IQ below 70. Current or past head trauma with loss of consciousness longer than 5 minutes, current or past neurological or somatic disorders potentially affecting the structure or functions of the brain, current or past alcohol dependence as well as polysubstance dependence within the past six months are other general exclusion criteria.

Individuals are included in the recent onset of psychosis (ROP) study group, if they fulfil the DSM-IV-TR criteria for affective and non-affective psychotic episode lifetime and if the psychotic episode is present within the past three months. Another ROP study group-specific inclusion criterion is that the onset of psychosis occurred within the past 24 months. ROP study group-specific exclusion criterion is antipsychotic medication for longer than 90 days (cumulative number of days) at or above minimum dosage of the 1st episode psychosis range of DGPPN (German Association for Psychiatry, Psychotherapy and Psychosomatics) S3 guideline.

The clinical evaluation of psychopathological symptom severity, as well as the clinical assessments of clinical outcomes, social and role functioning, have been conducted by trained physicians or clinical psychologist and interrater reliability tests were performed to minimize any site or rater effect. The neuropsychological assessments with 12 paper-pencil and tablet-based tests adapted for the PRONIA consortium (Supplementary Table 1) were administered by trained personnel. All neuropsychological tests were instructed by trained clinical psychologists and lasted for about 2 hours on average. All participants underwent the same tasks in the same order and were guided by standardized instructions. In language-dependent tasks, English speaking participants or participants who were insufficient in German were evaluated based on test material in English.

**2. Scaling Data**

Since the clustering results depend on the (dis)similarity between two observations, scaling the data is necessary to have consistent distance measures and to exclude the effect of different ranges in data. We scaled the data to a 0 - 1 range to avoid inconsistency by detecting (dis)similarities among observations due to different range of PANSS (rated on a scale from 1: not present to 7: extremely severe) and SANS (rated on a scale from 0: not at all to 6: severe). After subtracting 1 from PANSS items (PANSS score - 1) to get a range of PANSS scale from 0: not present to 6: extremely severe, we scaled data to a 0 - 1 range from both PANSS score and SANS score with the following function in RStudio: scaletorange = function(x){(x - min(x))/diff(range(x))} . Therefore, the minimum; not present in the SANS and the PANSS scale were treated mathematically as equal in the algorithms. This scaling provided the clustering algorithms with better detection of similar patterns as well as a better differentiation of dissimilarity between the pairs of observations in the given clinical dataset.

**3. The decision for k-means clustering and re-evaluation of the clustering stability**

Even though clustering algorithms are useful computational methods to recognize multivariate patterns in a given data, these are not straightforward approaches. There are important steps to get robust, replicable and generalizable clustering solutions with clinical utility. In general, we explored the clinical heterogeneity in psychosis in a data-driven manner. First, we applied *ClValid* (Burock et al., 2008) package for three algorithms; k-means, hierarchical and partitioning around medoids. Hierarchical clustering algorithms were tested for two different linkages: average and ward. As the results (Supplementary Table 2 and 3) show, k-means and hierarchical clustering using average linkage with 2 clusters were the most valid solutions.

Second, we retested these results with *NbClust* (Charrad et al., 2014) package if the optimal number, two is a robust solution. The *NbClust* package provided us with two possible solutions: hierarchical clustering using average linkage with four clusters and k-means with two clusters. We considered also the second optimal solutions; hierarchical clustering using average linkage with two clusters and hierarchical clustering using ward linkage with three clusters. These results are displayed in Supplementary Figure 1. We report the reason for the exclusion of these hierarchical algorithm solutions in the following section. Then we examined the stability and generalizability of the k-means solution with two clusters that we report in Supplementary Section 5 & 6. Thus, we tested many common indices for each step and majority rule showed two as the optimal number of clusters and k-means algorithm provided the most valid, stable and generalizable clustering solution.

**4. Exclusion the hierarchical clustering solutions**

The *ClValid* package comparing hierarchical clustering with average linkage and Ward linkage, k-means and partitioning around medoids resulted in two as the optimal numbers of clusters for hierarchical clustering using average linkage. We retested the possible optimal number of clusters with the *NbClust* package that provided us with different optimal numbers for average and ward linkage. These solutions for the hierarchical clustering were re-evaluated with the figure for silhouette width as well as the cophenetic correlation and this clustering method was not selected for further analysis due to low cophenetic correlation values, unbalanced cluster size and amount of wrongly clustered subjects showing negative silhouette width that reduces the clinical relevance of those results. (Supplementary Figure 2)

**5. The re-evaluation of the cluster stability**

The *ClusterStability* (Lord et al., 2017) package was used to retest the robustness of the clustering solution with the k-means algorithm. This R package considers the common validity measures; the Calinski–Harabasz (Caliński & Harabasz, 1974), Silhouette (Caliński & Harabasz, 1974), Dunn (Dunn†, 1974) and Davies–Bouldin (Davies & Bouldin, 1979) measures and provides researchers with a global stability index (global ST-index) and an index for the stability of individual objects (individual ST-index) ranging from 0 to 1, where 1 indicates very strong stability. In the presented study, we applied *ClusterStability* with 500 replications for the k-means algorithms with cluster numbers of two. All global ST-indexes, as well as all individual ST-indexes, were 1. To illustrate the decreasing stability with varying optimal numbers of clusters, we displayed the outputs of global ST-indexes in Supplementary Figure 3.

**6. The generalizability of the cluster solution**

The *predict.strength* (Tibshirani & Walther, 2005) package is an implementation of cross-validation, a widely used generalization method in supervised machine learning algorithms, for a given unsupervised machine learning question. The principals of the *predict.strength* algorithm are n-fold random resampling and partitioning of observations in training and test sets through m iterations in a given data where the proportion of pair – observations falling into the same clusters is computed. Thibshirani et. Whalter compared 2-fold cross-validation with 5-fold, that did not increase the predictive strength performance, and they continued with 2-fold cross-validation for the further analyses in the original publication. (Tibshirani & Walther, 2005) They provided predicting strength values between 0.8 – 0.9 as optimal. Therefore, subjects were randomly resampled 500 times with a 2-fold cross-validation manner and the number of clusters with highest prediction strength over the cut off value 0.80 was chosen as an optimal number of clusters in the presented study (Supplementary Figure 4).

**7. Comparison of psychopathological subdomains of clusters**

In order to test whether our clustering solution was associated with global and syndromal disease severity, we did group level statistical comparison using the PANSS and SANS total scores, as well as each PANSS and SANS subscales between the identified FTD subgroups and reported the results showing the psychopathological surrogates of the clustering solution in the Supplementary Table 6. Notably, as reported in the Supplementary Table 6 the statistical comparison of PANSS total or subscale scores between two FTD subgroups did not differ from each other that indicates the specificity of these cluster solution for FTD. In contrast, the SANS scores showed a strong association with our FTD-informed patient subgroups that is in keeping with previous findings linking FTD, negative and cognitive symptom domains to poorer functioning in psychotic disorders. (Cacciotti-Saija et al., 2018; Gerritsen et al., 2019) The NIMH-MATRICS consensus statement previously highlighted five negative symptom domains; blunted affect, alogia, asociality, anhedonia and avolition for a more comprehensive examination of negative symptoms. (Kirkpatrick et al., 2006) The SANS covers a larger part of these defined negative symptoms, whereas the PANSS negative subscale does not incorporate negative symptoms such avolition and anhedonia. (Daniel, 2013; Garcia-Portilla et al., 2015) The different operationalization of the negative symptoms construct in the PANSS and SANS might explain the discrepancy in our results between PANSS negative subscale and SANS.

**8. The cluster solution specificity and sanity analyses**

To investigate the interaction between FTD-related symptoms; Conceptual Disorganization, Poverty of Content of Speech, Difficulty in Abstract Thinking, Increased Latency of Response, Poverty of Speech and FTD subgroups; FTD-High and FTD-Low, we ran factorial ANOVA (Jaccard & Jaccard, 1998) and reported the results in the Supplementary Table 7 and Supplementary Figure 6. The FTD-related symptoms and the FTD subgroups were entered in the factorial ANOVA to test the main effects of these symptoms and of these subgroups as well as their interaction with each other. Our findings showed a significant main effect of FTD subgroups (*p* < 0.001), a significant main effect of FTD-symptoms (*p* < 0.001) as well as a significant interaction between FTD-symptoms and FTD subgroups (*p* = 0.025).

Moreover, to corroborate the specificity of our clustering solution with FTD-related symptoms, we run the multi-step clustering protocol with PANSS and SANS items that are not related to FTD (Supplementary Figure 7). To exclude that the clustering algorithms provide us with a solution more prone to detect negative symptom pattern, we applied our clustering protocol using (i) 6 items from PANSS negative subscale without the item Difficulty in Abstract Thinking which has been used for the FTD-driven clustering solution (Supplementary Figure 8) and (ii) using all other 17 items from SANS excluding FTD-related symptoms; Poverty of Content of Speech, Increased Latency of Response and Poverty of Speech (Supplementary Figure 9). To test whether our clustering protocol recognizes a positive symptom pattern using 6 items from PANSS positive subscale without Conceptual Disorganization, we applied our clustering protocol to these variables. As reported in the Supplementary Figure 10, the clustering analysis using PANSS positive items without FTD-related Conceptual Disorganization as input has also provided a stable (global ST(stability)-index = 0.99) and highly generalizable (predict.strength value=0.88) two-cluster solution with k-means algorithm. We compared the original FTD-driven solution (n = 279; n_FTD-High = 75,_ n_FTD-Low = 204_) and the cluster solution driven by positive items without Conceptual Disorganization (n=279; n_cluster1= 128,_ n_cluster2 = 151_) in their size. We also compared the clustering assignment of each participant to observe the proportion of participants who have been assigned into Cluster 1 or Cluster 2. These comparisons showed that (i) the cluster solutions are different in their size and (ii) 149 / 279 participants have been assigned to other clusters. These results together with the results of the factorial ANOVA also strengthen that the clustering solutions are driven by the contribution of different psychopathological domains and that the symptoms entered to clustering algorithm as inputs matter.

To sum up, these sanity analyses showed us that our FTD-driven clustering solution was clinically valid and specific to FTD-related symptom severity. Analyses with non-FTD items from PANSS and SANS as well as their subscale showed less stable and less generalizable clustering solutions than the FTD-driven clustering solution.

**8. Comparison of neurocognitive performances corrected for years of education**

Comparisons of neurocognitive measures corrected for years of education between FTD subgroups showed significant differences in verbal and semantic fluency, verbal short-term memory and abstract reasoning (Supplementary Table 8). The WAIS-Vocabulary (p_fdr_ = 0.042, r = 0.129) and WAIS-Matrices (p_fdr_ = 0.048, r = 0.130) scores were lower in the FTD-High group than in the FTD-Low group. We found a similar pattern of results in the phonological verbal fluency (p_fdr_ = 0.009, r = 0.196) and semantic fluency (p_fdr_ = 0.012, r = 0.288) scores, as well as in the forward (p_fdr_ = 0.016, r = 0.159) digit span scores, i.e., FTD-High individuals always performed worse than FTD-Low group in these neurocognitive domains. The statistical significance was not observed in the backward (p_fdr_ = 0.108, r = 0.099) digit span scores.

**9. Supplementary Tables 1 - 7**

**Supplementary Table 1. The neurocognitive battery used in the PRONIA study in order of administration.** ^a^ test used for analysis, ^b^ revised version of the Hopkins’ Verbal Learning Test for the University of Turku.

| **The name of the neurocognitive assessment** | **The cognitive domains** | **Administration** |
| --- | --- | --- |
| Rey-Osterrieth Complex Figure (ROCF) (Gagnon et al., 2003; A M Hubley, 1996; Anita M Hubley & Tremblay, 2002; Osterrieth, 1944) | visuo-spatial construction;  visuo-spatial memory  (short- and long-term) | paper-pencil format with tablet support |
| Diagnostic Analysis of Non-Verbal Accuracy (DANVA-2-AF) (Nowicki Jr & Carton, 1993; S Nowicki, 2000; Stephen Nowicki & Duke, 1994) | social cognition | tablet-based |
| Auditory Digit Span  Forward & Backward trials (ADS-F&B) ^a,^ (Orsini et al., 1987; Wechsler, 2008) | verbal short-term memory  verbal working memory | auditory presentation of numbers by recorded (male) voice |
| Verbal Fluency, Phonological & Semantic trials (VF-P&S) ^a,^ (Borkowski et al., 1967; Harrison et al., 2000) | verbal fluency;  in a phonemic ('S'-words) and  in a semantic ('Animals') condition | named words were recorded and written down by an examiner |
| Rey Auditory Verbal Learning Test  (RAVLT)^b,^ (McMinn et al., 1988; Rey, 1964) | short-term verbal memory  long-term verbal memory | auditory presentation  of word list by recorded (male) voice |
| Trail Making Task, A & B trials (TMT-A&B) (Horton Jr. & Hartlage, 1994) | processing speed  sequencing  graphomotor capacity  visual attention  search ability and flexibility | paper-pencil format |
| Continuous Performance Test  Identical Pairs version (CPT-IP) (Bellani & Brambilla, 2008; Cornblatt et al., 1988, 1989) | selective visual attention  sustained visual attention | tablet-based |
| Self-Ordered Pointing Test (SOPT) (Gillett, 2007; Milner et al., 1985; Petrides & Milner, 1982) | short-term visuospatial memory short-term working memory | tablet-based |
| Digit Symbol Substitution Test (DSST) (Keefe et al., 2004; Wechsler, 2008) | sustained attention  working memory  processing speed | paper-pencil format |
| Salience Attribution Task (SAT-SV) (J P Roiser et al., 2009; Jonathan P Roiser et al., 2010, 2012) | explicit and implicit salience  adaptive and aberrant salience | tablet-based |
| Wechsler Adult Intelligence Scale (WAIS-III) ^a,^ (Wechsler, 2008) |  |  |
| *Vocabulary* | premorbid verbal intelligence | paper-pencil format |
| *Matrices* | visual processing and abstract reasoning | paper-pencil format |

**Supplementary Table 2.** The results from the *ClValid* package comparing algorithms; hierarchical using ward linkage, k-means and partitioning around medoids (pam) for internal validity and stability measures. Results in bold show the optimal clustering method represented with ^a^ and the optimal number of clusters represented with ^b^ for each measure.

| **Clustering**  **Methods** | **No. of clusters** | **Stability measures** | | | | **Internal validity measures** | | |
| --- | --- | --- | --- | --- | --- | --- | --- | --- |
|  |  | **APN** | **AD** | **ADM** | **FOM** | **Connectivity** | **Dunn** | **Silhouette** |
| **Hierarchical** | **2 ^b^** | 0.2859 | 0.5916 | 0.2264 | 0.2169 | **42.4440 ^a^** | 0.0894 | 0.3354 |
|  | 3 | 0.4275 | 0.5469 | 0.2560 | 0.2121 | 55.1198 | 0.1072 | 0.3492 |
|  | 4 | 0.3615 | 0.4897 | 0.2217 | 0.2082 | 65.0385 | 0.1072 | 0.2116 |
|  | 5 | 0.3970 | 0.4676 | 0.2181 | 0.2061 | 86.2671 | 0.1072 | 0.2290 |
|  | 6 | 0.4187 | 0.4514 | 0.2271 | 0.2049 | 103.6698 | 0.1072 | 0.2471 |
|  | 7 | 0.4398 | 0.4384 | 0.2368 | 0.2011 | 108.6286 | 0.1162 | 0.2568 |
|  | 8 | 0.4059 | 0.4219 | 0.2338 | 0.1976 | 110.9214 | 0.1162 | 0.3027 |
|  | 9 | 0.3860 | 0.4061 | 0.2263 | 0.1956 | 125.0655 | 0.1162 | 0.3114 |
|  | **10 ^b^** | 0.3469 | 0.3875 | 0.2101 | **0.1903 ^a^** | 129.9125 | 0.1162 | 0.3364 |
| **k-means** | **2 ^b^** | **0.1081^a^** | 0.5312 | **0.0813 ^a^** | 0.2071 | 46.7008 | **0.1516 ^a^** | **0.4586 ^a^** |
|  | 3 | 0.1832 | 0.4873 | 0.1224 | 0,2030 | 66.0560 | 0.1072 | 0.3813 |
|  | 4 | 0.2687 | 0.4668 | 0.1883 | 0.2037 | 89.5635 | 0.1072 | 0.3469 |
|  | 5 | 0.3620 | 0.4611 | 0.2128 | 0.2044 | 99.2310 | 0.1187 | 0.3623 |
|  | 6 | 0.3977 | 0.4435 | 0.2192 | 0.1999 | 111.0052 | 0.1187 | 0.3652 |
|  | 7 | 0.4216 | 0.4299 | 0.2288 | 0.1989 | 119.0147 | 0.1280 | 0.3715 |
|  | 8 | 0.3651 | 0.4095 | 0.2136 | 0.1965 | 124.5056 | 0.1280 | 0.3237 |
|  | 9 | 0.3716 | 0.3974 | 0.2116 | 0.1950 | 122.3010 | 0.1280 | 0.3356 |
|  | 10 | 0.3191 | 0.3697 | 0.1845 | 0.1903 | 133.2766 | 0.1348 | 0.3613 |
| **pam** | 2 | 0.1495 | 0.5314 | 0.1011 | 0.2128 | 66.1817 | 0.0894 | 0.4125 |
|  | 3 | 0.1802 | 0.4816 | 0.1123 | 0.2036 | 75.1012 | 0.0971 | 0.3354 |
|  | 4 | 0.3385 | 0.4734 | 0.1868 | 0.2021 | 97.5560 | 0.1072 | 0.3558 |
|  | 5 | 0.3097 | 0.4480 | 0.1755 | 0.1995 | 102.8889 | 0.1072 | 0.2725 |
|  | 6 | 0.2850 | 0.4230 | 0.1735 | 0.1984 | 110.4143 | 0.1072 | 0.2899 |
|  | 7 | 0.3268 | 0.4150 | 0.1908 | 0.1954 | 135.4651 | 0.1091 | 0.2938 |
|  | 8 | 0.3783 | 0.4099 | 0.2110 | 0.1939 | 120.7849 | 0.1222 | 0.3104 |
|  | 9 | 0.3217 | 0.3921 | 0.2054 | 0.1931 | 134.3079 | 0.1091 | 0.3345 |
|  | **10 ^b^** | 0.2825 | **0.3669 ^a^** | 0.1711 | 0.1907 | 133.4821 | 0.1022 | 0.3515 |

**Supplementary Table 3.** The results from the *ClValid* package comparing algorithms; hierarchical using average linkage, k-means and partitioning around medoids (pam) for internal validity and stability measures. Results in bold show the optimal clustering method represented with ^a^ and the optimal number of clusters represented with ^b^ for each measure.

| **Clustering**  **Methods** | **No. of clusters** | **Stability measures** | | | | **Internal validity measures** | | |
| --- | --- | --- | --- | --- | --- | --- | --- | --- |
|  |  | **APN** | **AD** | **ADM** | **FOM** | **Connectivity** | **Dunn** | **Silhouette** |
| **Hierarchical** | **2 ^b^** | **0.0181 ^a^** | 0.6283 | **0.0337 ^a^** | 0.2287 | **7.3155 ^a^** | **0.3078 ^a^** | **0.5420 ^a^** |
|  | 3 | 0.1290 | 0.6124 | 0.1135 | 0.2229 | 29.2960 | 0.1451 | 0.3660 |
|  | 4 | 0.1341 | 0.5644 | 0.1626 | 0.2180 | 57.2714 | 0.1591 | 0.4047 |
|  | 5 | 0.1674 | 0.5383 | 0.1530 | 0.2086 | 61.6738 | 0.1715 | 0.3763 |
|  | 6 | 0.1732 | 0.5323 | 0.1507 | 0.2068 | 62.3905 | 0.1715 | 0.3694 |
|  | 7 | 0.2273 | 0.5248 | 0.1633 | 0.2046 | 65.2083 | 0.1715 | 0.3613 |
|  | 8 | 0.3137 | 0.5247 | 0.1952 | 0.2003 | 67.8194 | 0.1715 | 0.3609 |
|  | 9 | 0.2664 | 0.4857 | 0.1733 | 0.1982 | 83.8452 | 0.1980 | 0.3495 |
|  | 10 | 0.2770 | 0.4820 | 0.1738 | 0.1973 | 83.9563 | 0.1980 | 0.3440 |
| **k-means** | 2 | 0.0941 | 0.5314 | 0.0736 | 0.2072 | 46.7008 | 0.1516 | 0.4586 |
|  | 3 | 0.1664 | 0.4973 | 0.1393 | 0,2052 | 67.6091 | 0.1072 | 0.3911 |
|  | 4 | 0.2332 | 0.4776 | 0.1647 | 0.2069 | 85.4873 | 0.1187 | 0.4029 |
|  | 5 | 0.2584 | 0.4594 | 0.1722 | 0.2007 | 122.3821 | 0.1313 | 0.3877 |
|  | 6 | 0.3147 | 0.4537 | 0.1990 | 0.2001 | 122.8413 | 0.1313 | 0.3826 |
|  | 7 | 0.3047 | 0.4345 | 0.1901 | 0.1982 | 131.0464 | 0.1313 | 0.3688 |
|  | 8 | 0.3139 | 0.4205 | 0.1984 | 0.1971 | 139.2278 | 0.1400 | 0.3647 |
|  | 9 | 0.3634 | 0.4273 | 0.2149 | 0.1965 | 134.9710 | 0.1601 | 0.3570 |
|  | 10 | 0.3621 | 0.4066 | 0.2076 | 0.1914 | 153.7004 | 0.1690 | 0.3653 |
| **pam** | 2 | 0.1495 | 0.5314 | 0.1011 | 0.2128 | 66.1817 | 0.0894 | 0.4125 |
|  | 3 | 0.1802 | 0.4816 | 0.1123 | 0.2036 | 75.1012 | 0.0971 | 0.3354 |
|  | 4 | 0.3385 | 0.4734 | 0.1868 | 0.2021 | 97.5560 | 0.1072 | 0.3558 |
|  | 5 | 0.3097 | 0.4480 | 0.1755 | 0.1995 | 102.8889 | 0.1072 | 0.2725 |
|  | 6 | 0.2850 | 0.4230 | 0.1735 | 0.1984 | 110.4143 | 0.1072 | 0.2899 |
|  | 7 | 0.3268 | 0.4150 | 0.1908 | 0.1954 | 135.4651 | 0.1091 | 0.2938 |
|  | 8 | 0.3783 | 0.4099 | 0.2110 | 0.1939 | 120.7849 | 0.1222 | 0.3104 |
|  | 9 | 0.3217 | 0.3921 | 0.2054 | 0.1931 | 134.3079 | 0.1091 | 0.3345 |
|  | **10 ^b^** | 0.2825 | **0.3669 ^a^** | 0.1711 | **0.1907 ^a^** | 133.4821 | 0.1022 | 0.3515 |

**Supplementary Table 4:** Represents the results of the Shapiro – Wilk normality test for each statistical comparison, *P* values are shown only with 4 decimals.

|  | **FTD - High** | | | **FTD - Low** | | |
| --- | --- | --- | --- | --- | --- | --- |
|  | **w** | | ***P* value** | | **w** | ***P* value** |
| Age | 0.9401 | 0.002 | | | 0.9519 | < 0.001 |
| Education year | 0.9385 | 0.001 | | | 0.9766 | 0.002 |
| GF Social highest lifetime | 0.8425 | < 0.001 | | | 0.8783 | < 0.001 |
| GF Social highest in past year | 0.9360 | 0.001 | | | 0.9253 | < 0.001 |
| GF Social lowest in past year | 0.9515 | 0.008 | | | 0.9505 | < 0.001 |
| GF Social current | 0.9441 | 0.003 | | | 0.9465 | < 0.001 |
| GF Role highest lifetime | 0.8144 | < 0.001 | | | 0.8932 | < 0.001 |
| GF Role highest in past year | 0.9475 | 0.004 | | | 0.8695 | < 0.001 |
| GF Role lowest in past year | 0.9414 | 0.002 | | | 0.9531 | < 0.001 |
| GF Role current | 0.9514 | 0.007 | | | 0.9480 | < 0.001 |
| WAIS – premorbid verbal intelligence | 0.9758 | 0.193 | | | 0.9821 | 0.018 |
| WAIS - Matrices | 0.9767 | 0.233 | | | 0.9669 | < 0.001 |
| Phonological Verbal Fluency | 0.9534 | 0.011 | | | 0.9907 | 0.253 |
| Semantic Verbal Fluency | 0.9813 | 0.390 | | | 0.9930 | 0.498 |
| Forward Digit Span | 0.9620 | 0.031 | | | 0.9719 | 0.001 |
| Backward Digit Span | 0.9535 | 0.010 | | | 0.9643 | < 0.001 |
| PANSS Total | 0.9757 | 0.166 | | | 0.9803 | 0.006 |
| PANSS Positive | 0.9534 | 0.008 | | | 0.9771 | 0.002 |
| PANSS Negative | 0.9141 | < 0.001 | | | 0.9171 | < 0.001 |
| PANSS General | 0.9594 | 0.018 | | | 0.9742 | 0.001 |
| SANS Total | 0.9648 | 0.037 | | | 0.9467 | < 0.001 |
| SANS Blunting | 0.9233 | < 0.001 | | | 0.8112 | < 0.001 |
| SANS Alogia | 0.9007 | < 0.001 | | | 0.6786 | < 0.001 |
| SANS Avolition | 0.8930 | < 0.001 | | | 0.8926 | < 0.001 |
| SANS Anhedonia | 0.8931 | < 0.001 | | | 0.9002 | < 0.001 |
| SANS Attention | 0.8885 | < 0.001 | | | 0.7884 | < 0.001 |

**Supplementary Table 5:** Represents the number of subjects with missing values per cluster.

|  | **FTD - High** | **FTD - Low** | ***P*-value** |  |  |
| --- | --- | --- | --- | --- | --- |
| Education | 0 | 1 | 1 |  |  |
| WAIS premorbid verbal Intelligence | 4 | 18 | 0.502 |  |  |
| WAIS Matrices | 6 | 21 | 0.762 |  |  |
| Phonological verbal fluency | 4 | 12 | 1 |  |  |
| Semantic verbal fluency | 5 | 12 | 1 |  |  |
| Forward digit span | 3 | 11 | 0.895 |  |  |
| Backward digit span | 3 | 11 | 0.895 |  |  |

**Supplementary Table 6:** Comparison of two FTD subgroups in PANSS and SANS.

| **Characteristics** | **Formal Thought Disorder-related symptom severity** | | | |
| --- | --- | --- | --- | --- |
|  | **High** | **Low** | ***p_fdr_* value** | ***p* value** |
| PANSS at baseline |  |  |  |  |
| Total, median | 65.5 | 67 | 0.780 | 0.7780 |
| Positive, median | 16 | 18 | 0.392 | 0.275 |
| Negative, median | 16 | 14 | 0.438 | 0.351 |
| General, median | 32 | 34 | 0.506 | 0.456 |
| SANS at baseline |  |  |  |  |
| Total, median | 46.5 | 16 | < 0.001 | < 0.001 |
| Blunting, median | 15 | 3 | < 0.001 | < 0.001 |
| Alogia, median | 9 | 0 | < 0.001 | < 0.001 |
| Avolition, median | 7 | 4 | < 0.001 | < 0.001 |
| Anhedonia, median | 11 | 6 | < 0.001 | < 0.001 |
| Attention, median | 4 | 0 | < 0.001 | < 0.001 |

**Supplementary Table 7:** Representation of results of factorial ANOVA using scaled FTD-related Symptom Severity data**.**

|  | | **Df** | **Sum Sq** | **Mean Sq** | **F** |  | **Pr(>F)** | |
| --- | --- | --- | --- | --- | --- | --- | --- | --- |
|  |  |  |  |  |  |  |  |  |
| Clusters | | 1 | 30.52 | 30.519 | 865.356 |  | < 0.001 *** |  |
| Features | | 4 | 1.13 | 0.283 | 8.013 |  | < 0.001 *** |  |
| Clusters: Features | | 4 | 0.39 | 0.098 | 2.786 |  | 0.025 * |  |
| Residuals | | 1385 | 48.85 | 0.035 |  |  |  |  |

**Supplementary Table 8:** Neurocognition Differences corrected for years of education in Individuals with Recent-Onset Psychosis

| **Characteristics** | **Formal Thought Disorder related symptom severity** | | | |
| --- | --- | --- | --- | --- |
|  | **Low** | **High** | ***p* value** | ***p_fdr_* value** |
| Neurocognition at baseline |  |  |  |  |
| WAIS - premorbid verbal intelligence,  median | 10 | 9 | 0.078 | 0.042 |
| WAIS - Matrices, median | 10 | 9 | 0.040 | 0.048 |
| Phonological Verbal Fluency, median | 13 | 11 | 0.001 | 0.009 |
| Semantic Verbal Fluency, median | 21 | 16 | 0.004 | 0.012 |
| Forward Digit Span, median | 9 | 8 | 0.008 | 0.016 |
| Backward Digit Span, median | 6 | 6 | 0.108 | 0.108 |

**10. Supplementary Figures 1 - 10**

**Supplementary Figure 1:** The results from *NbClust* package showing the different optimal number of clusters.


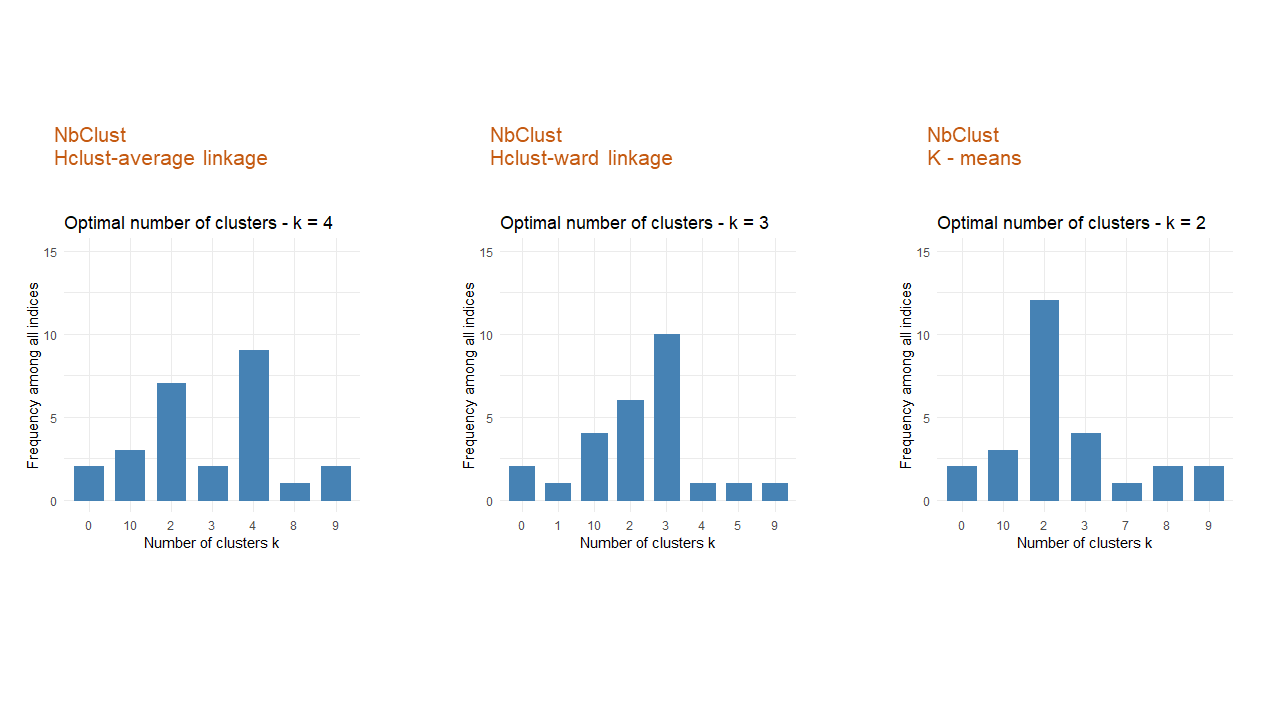


**Supplementary Figure 2:** The quality check for the solutions with hierarchical clustering algorithms**.**


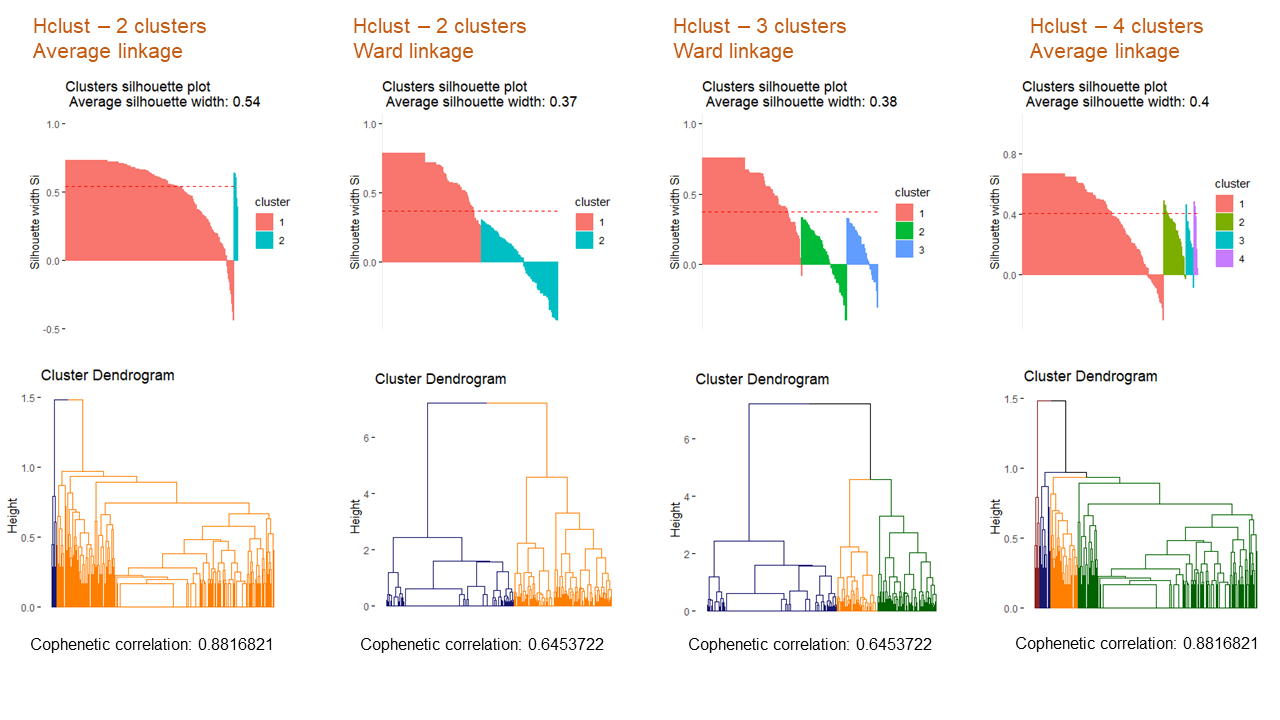


**Supplementary Figure 3:** Representation of the decreasing stability with an increasing number of clusters based on four stability indices; ch:Calinski–Harabasz, db: Davies–Bouldin, sil: Silhouette.


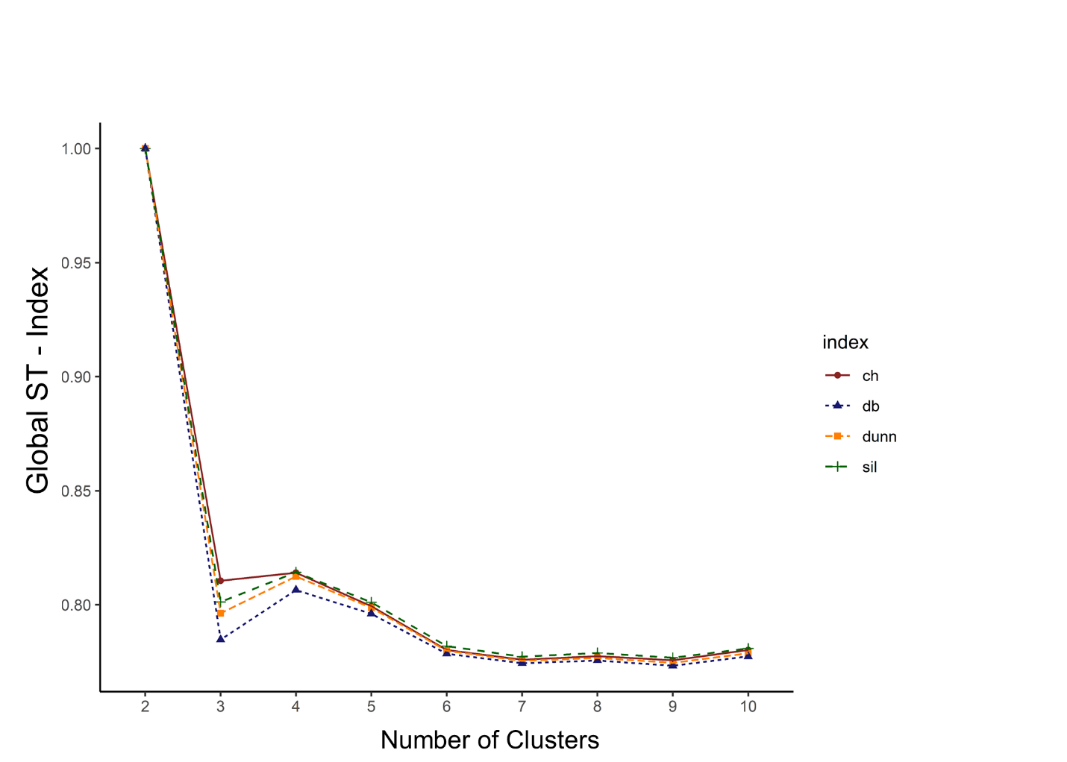


**Supplementary Figure 4:** Representation of the settings and results of the generalizability test with *predict.strength* package


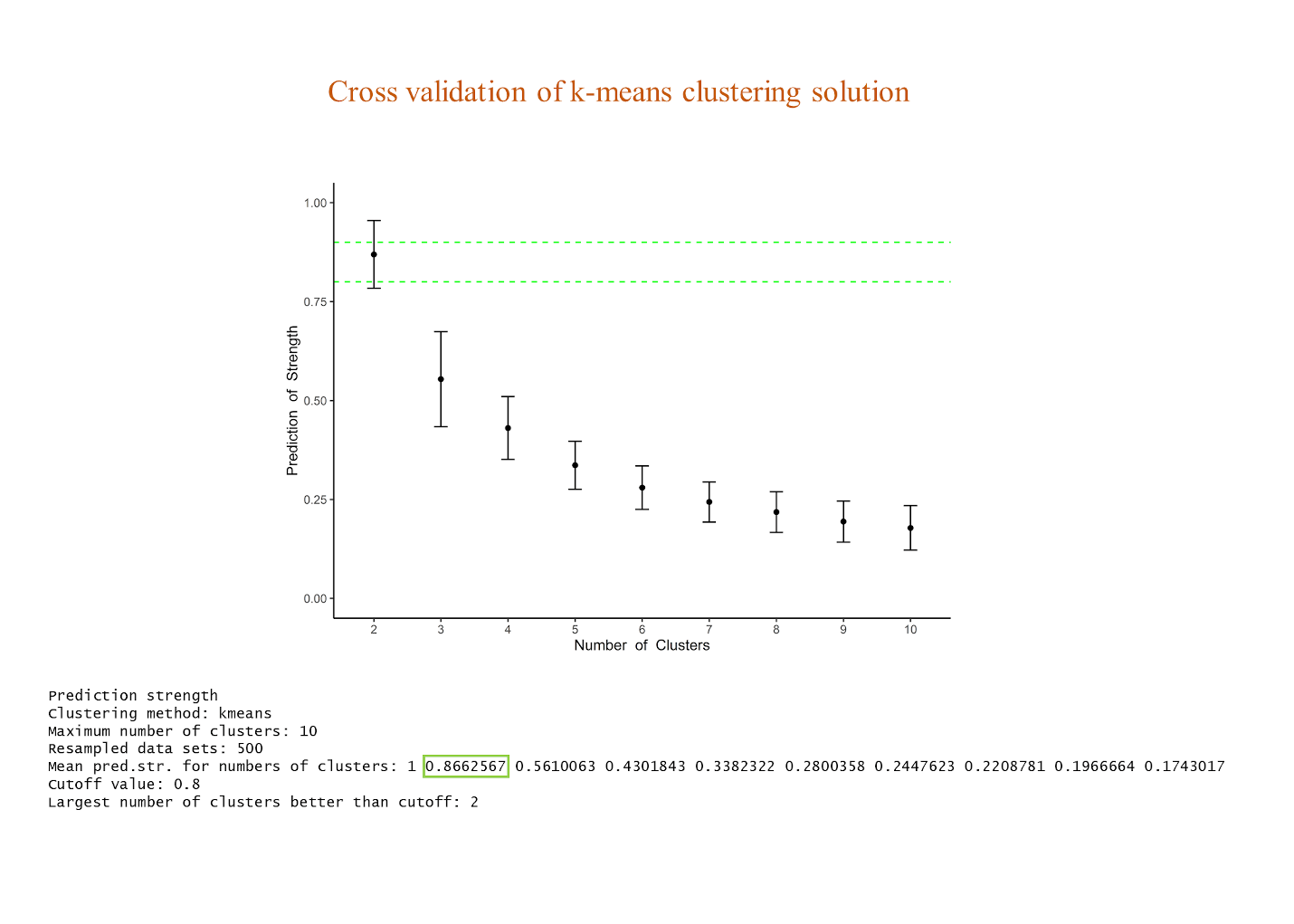


**Supplementary Figure 5:** Representation of the age and sex distribution of study participants with a recent-onset psychosis from the PRONIA cohort.

**
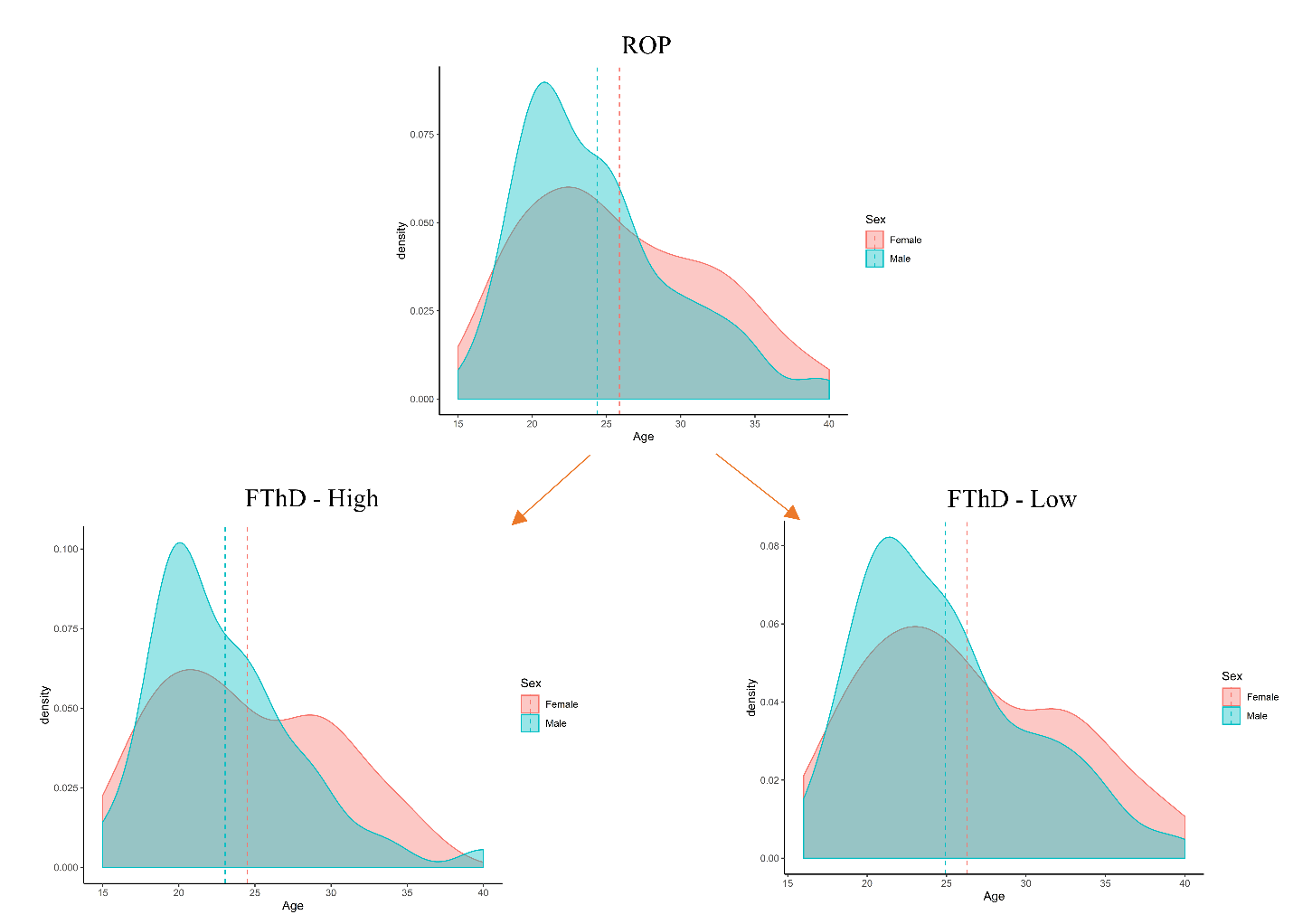
**

**Supplementary Figure 6:** Representation of the predicted values of symptom severity in FTD-clusters; results of factorial ANOVA using scaled FTD-related Symptom Severity data.

**
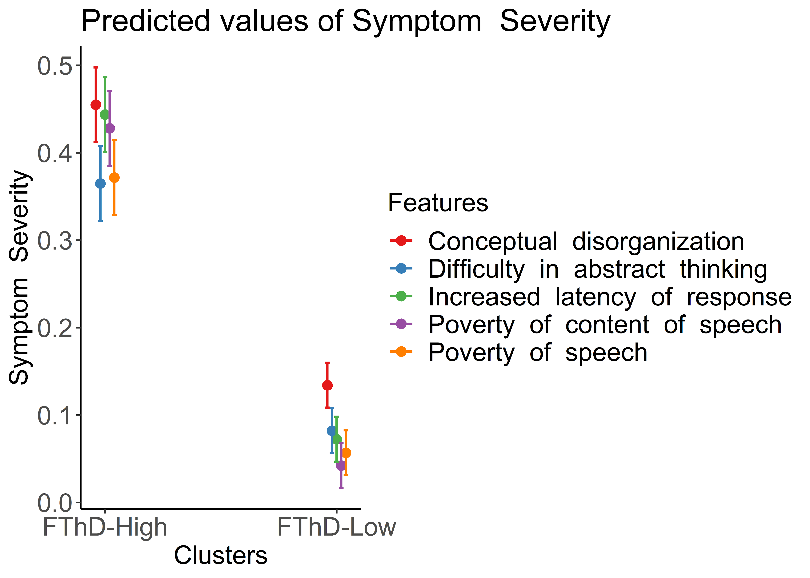
**

**Supplementary Figure 7:** Representation of the multi-step clustering solutions using PANSS and SANS items that are not related to FTD as input variables.

**
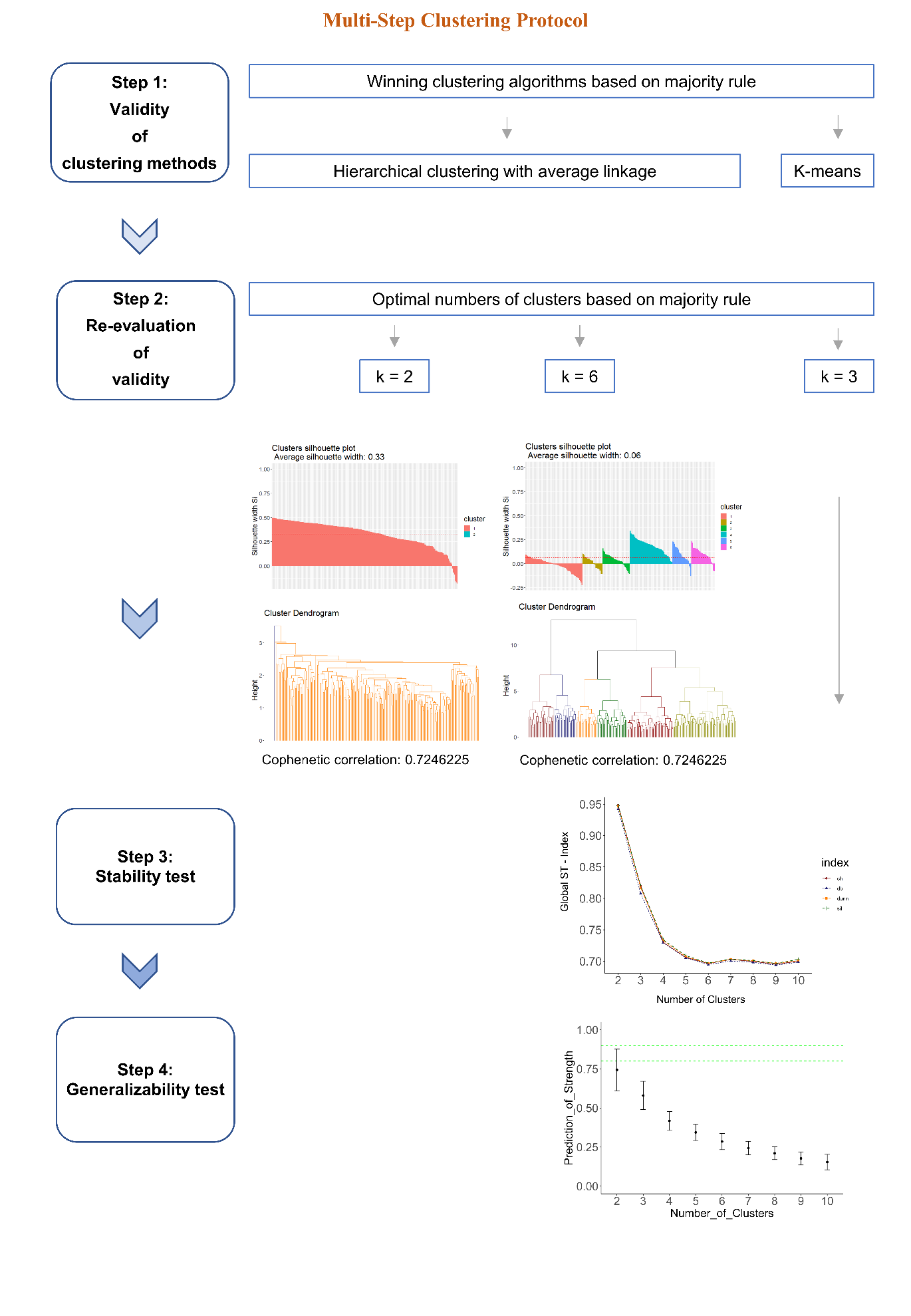
**

**Supplementary Figure 8:** Representation of the multi-step clustering solutions using PANSS negative subscale items that are not related to FTD as input variables.

**
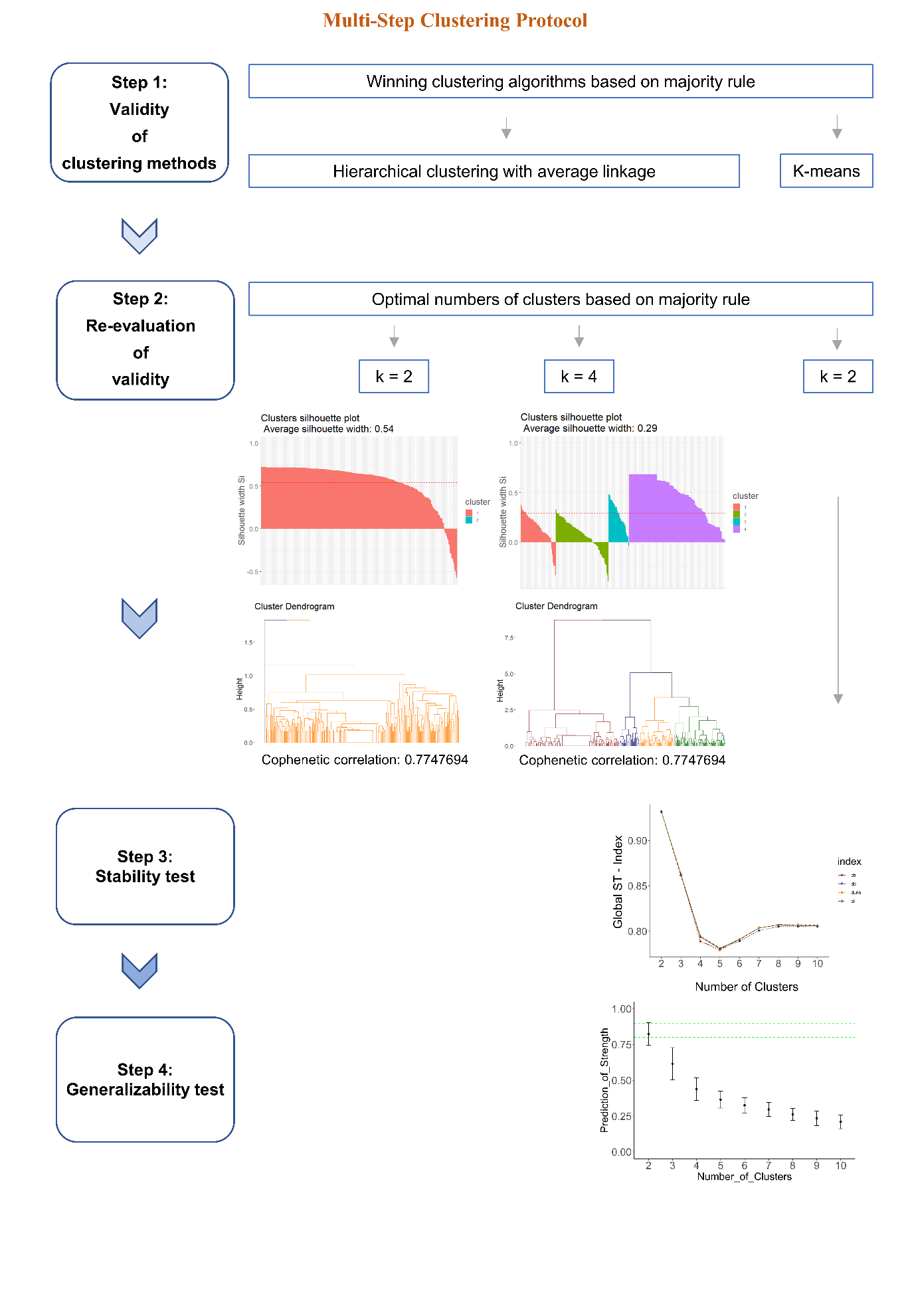
**

**Supplementary Figure 9:** Representation of the multi-step clustering solutions using SANS items that are not related to FTD as input variables.

**
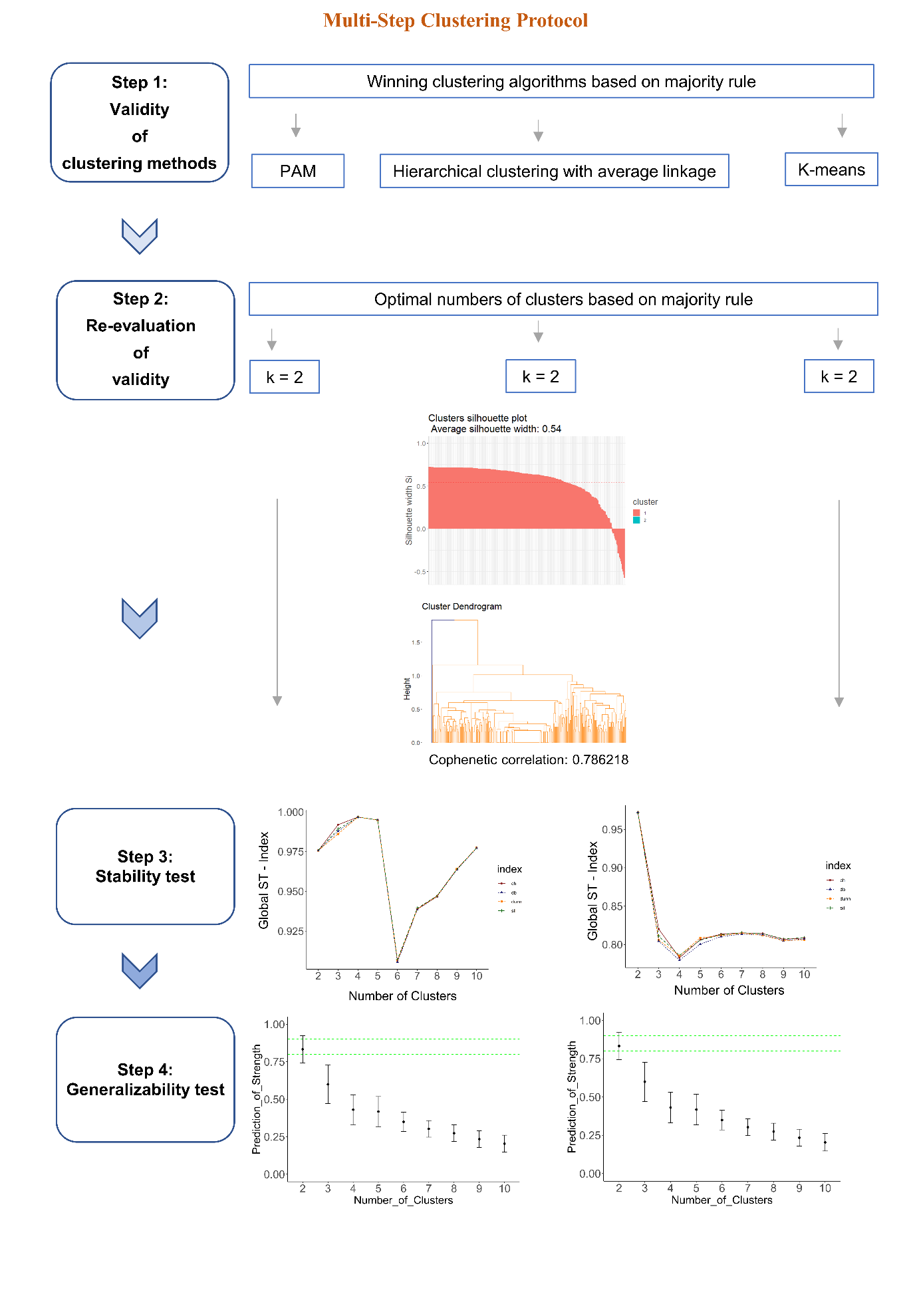
**

**Supplementary Figure 10:** Representation of the multi-step clustering solutions using PANSS positive subscale items that are not related to FTD as input variables.**
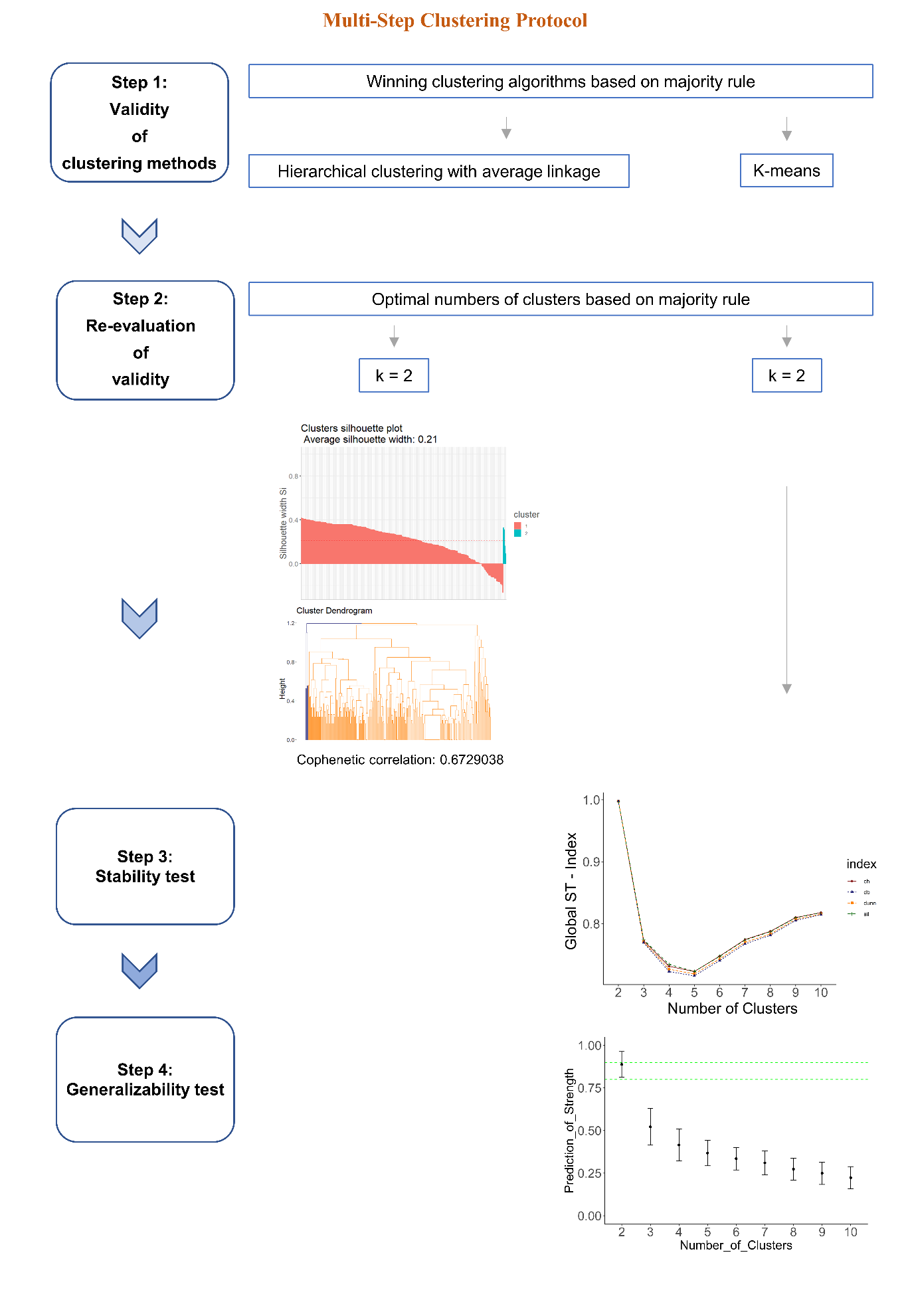
**

**References**

Bellani, M., & Brambilla, P. (2008). The use and meaning of the continuous performance test in schizophrenia. *Epidemiology and Psychiatric Sciences*, *17*(3), 188–191.

Borkowski, J. G., Benton, A. L., & Spreen, O. (1967). Word fluency and brain damage. *Neuropsychologia*, *5*(2), 135–140.

Burock, G., Pihur, V., Datta, S., & Datta, S. (2008). clValid: An R Package for Cluster Validation. *Journal of Statictical Software*, *25*(4).

Cacciotti-Saija, C., Langdon, R., Ward, P. B., Hickie, I. B., & Guastella, A. J. (2018). Clinical symptoms predict concurrent social and global functioning in an early psychosis sample. *Early Intervention in Psychiatry*, *12*(2), 177–184.

Caliński, T., & Harabasz, J. (1974). A dendrite method for cluster analysis. *Communications in Statistics-Theory and Methods*, *3*(1), 1–27.

Charrad, M., Ghazzali, N., Boiteau, V., & Niknafs, A. (2014). NbClust: An R Package for Determining the Relevant Number of Clusters in a Data Set. *Journal of Statistical Software*.

Cornblatt, B. A., Lenzenweger, M. F., & Erlenmeyer-Kimling, L. (1989). The continuous performance test, identical pairs version: II. Contrasting attentional profiles in schizophrenic and depressed patients. *Psychiatry Research*, *29*(1), 65–85.

Cornblatt, B. A., Risch, N. J., Faris, G., Friedman, D., & Erlenmeyer-Kimling, L. (1988). The Continuous Performance Test, identical pairs version (CPT-IP): I. New findings about sustained attention in normal families. *Psychiatry Research*, *26*(2), 223–238.

Daniel, D. G. (2013). Issues in Selection of Instruments to Measure Negative Symptoms. *Schizophrenia Research*, *150*(2–3), 343–345.

Davies, D. L., & Bouldin, D. W. (1979). A cluster separation measure. *IEEE Transactions on Pattern Analysis and Machine Intelligence*, *2*, 224–227.

Dunn†, J. C. (1974). Well-Separated Clusters and Optimal Fuzzy Partitions. *Journal of Cybernetics*, *4*(1), 95–104.

Gagnon, M., Awad, N., Mertens, V. B., & Messier, C. (2003). Comparing the Rey and Taylor Complex Figures: A Test-Retest Study in Young and Older Adults. *Journal of Clinical and Experimental Neuropsychology*, *25*(6), 878–890. https://doi.org/10.1076/jcen.25.6.878.16480

Garcia-Portilla, M. P., Garcia-Alvarez, L., Saiz, P. A., Al-Halabi, S., Bobes-Bascaran, M. T., Bascaran, M. T., Muñiz, J., & Bobes, J. (2015). Psychometric evaluation of the negative syndrome of schizophrenia. *European Archives of Psychiatry and Clinical Neuroscience*, *265*(7), 559–566.

Gerritsen, C., Maheandiran, M., Lepock, J., Ahmed, S., Kiang, M., Bagby, R. M., & Mizrahi, R. (2019). Negative symptoms in the clinical high‐risk state for psychosis: Connection with cognition and primacy in impacting functioning. *Early Intervention in Psychiatry*, *June*, eip.12843.

Gillett, R. (2007). Assessment of working memory performance in self-ordered selection tests. *Cortex*, *43*(8), 1047–1056.

Harrison, J. E., Buxton, P., Husain, M., & Wise, R. (2000). Short test of semantic and phonological fluency: Normal performance, validity and test‐retest reliability. *British Journal of Clinical Psychology*, *39*(2), 181–191.

Horton Jr., A. M., & Hartlage, L. C. (1994). The Halstead-Reitan neuropsychology test battery: Theory and clinical interpretation second edition. *Archives of Clinical Neuropsychology*, *9*(3), 289–290.

Hubley, A M. (1996). Modification of the Taylor Complex Figure: a comparable figure to the Rey-Osterrieth Figure? Edgeworth Series in Quantitative Behavioural. *Sci., Pap. No. ESQBS*, 96–97.

Hubley, Anita M, & Tremblay, D. (2002). Comparability of Total Score Performance on the Rey–Osterrieth Complex Figure and a Modified Taylor Complex Figure. *Journal of Clinical and Experimental Neuropsychology*, *24*(3), 370–382. https://doi.org/10.1076/jcen.24.3.370.984

Jaccard, J., & Jaccard, J. (1998). *Interaction effects in factorial analysis of variance* (Issue 118). Sage.

Keefe, R. S. E., Goldberg, T. E., Harvey, P. D., Gold, J. M., Poe, M. P., & Coughenour, L. (2004). The Brief Assessment of Cognition in Schizophrenia: reliability, sensitivity, and comparison with a standard neurocognitive battery. *Schizophrenia Research*, *68*(2–3), 283–297.

Kirkpatrick, B., Fenton, W. S., Carpenter, W. T., & Marder, S. R. (2006). The NIMH-MATRICS consensus statement on negative symptoms. *Schizophrenia Bulletin*, *32*(2), 214–219.

Koutsouleris, N., Kambeitz-Ilankovic, L., Ruhrmann, S., Rosen, M., Ruef, A., Dwyer, D. B., Paolini, M., Chisholm, K., Kambeitz, J., Haidl, T., Schmidt, A., Gillam, J., Schultze-Lutter, F., Falkai, P., Reiser, M., Riecher-Rössler, A., Upthegrove, R., Hietala, J., Salokangas, R. K. R., … Consortium, for the P. (2018). Prediction Models of Functional Outcomes for Individuals in the Clinical High-Risk State for Psychosis or With Recent-Onset Depression: A Multimodal, Multisite Machine Learning AnalysisPrediction Models of Functional Outcomes for the Clinical High-Risk St. *JAMA Psychiatry*, *75*(11), 1156–1172.

Lord, E., Willems, M., Lapointe, F. J., & Makarenkov, V. (2017). Using the stability of objects to determine the number of clusters in datasets. *Information Sciences*, *393*, 29–46.

McMinn, M. R., Wiens, A. N., & Crossen, J. R. (1988). Rey Auditory-Verbal Learning Test: Development of norms for healthy young adults. *The Clinical Neuropsychologist*, *2*(1), 67–87.

Milner, B., Petrides, M., & Smith, M. L. (1985). Frontal lobes and the temporal organization of memory. *Human Neurobiology*, *4*(3), 137–142.

Nowicki Jr, S., & Carton, J. (1993). The measurement of emotional intensity from facial expressions. *The Journal of Social Psychology*, *133*(5), 749–750.

Nowicki, S. (2000). Manual for the receptive tests of the Diagnostic Analysis of Nonverbal Accuracy 2. *Atlanta, GA: Department of Psychology, Emory University*.

Nowicki, Stephen, & Duke, M. P. (1994). Individual differences in the nonverbal communication of affect: The Diagnostic Analysis of Nonverbal Accuracy Scale. *Journal of Nonverbal Behavior*, *18*(1), 9–35.

Orsini, A., Grossi, D., Capitani, E., Laiacona, M., Papagno, C., & Vallar, G. (1987). Verbal and spatial immediate memory span: normative data from 1355 adults and 1112 children. *The Italian Journal of Neurological Sciences*, *8*(6), 537–548.

Osterrieth, P. A. (1944). Le test de copie d’une figure complexe; contribution à l’étude de la perception et de la mémoire. [Test of copying a complex figure; contribution to the study of perception and memory.]. *Archives de Psychologie*, *30*, 206–356.

Petrides, M., & Milner, B. (1982). Deficits on subject-ordered tasks after frontal-and temporal-lobe lesions in man. *Neuropsychologia*, *20*(3), 249–262.

Rey, A. (1964). L’Examen Clinique en Psychologie L’Examen Clinique en Psychologie. *Paris: Presses Universitaires de France*.

Roiser, J P, Stephan, K. E., Den Ouden, H. E. M., Barnes, T. R. E., Friston, K. J., & Joyce, E. M. (2009). Do patients with schizophrenia exhibit aberrant salience? *Psychological Medicine*, *39*(2), 199–209.

Roiser, Jonathan P, Howes, O. D., Chaddock, C. A., Joyce, E. M., & McGuire, P. (2012). Neural and behavioral correlates of aberrant salience in individuals at risk for psychosis. *Schizophrenia Bulletin*, *39*(6), 1328–1336.

Roiser, Jonathan P, Stephan, K. E., den Ouden, H. E. M., Friston, K. J., & Joyce, E. M. (2010). Adaptive and aberrant reward prediction signals in the human brain. *Neuroimage*, *50*(2), 657–664.

Tibshirani, R., & Walther, G. (2005). Cluster validation by prediction strength. *Journal of Computational and Graphical Statistics*, *14*(3), 511–528.

Wechsler, D. (2008). Wechsler adult intelligence scale–Fourth Edition (WAIS–IV). *San Antonio, TX: NCS Pearson*, *22*, 498.
